# Scientific production on health inequalities using the Brazilian Information System on Live Births: a scoping review

**DOI:** 10.1590/0102-311XEN197425

**Published:** 2026-07-31

**Authors:** Francine dos Santos Costa, Luiza Eunice Sá da Silva, Janaína Calu Costa, Cauane Blumenberg, Luis Paulo Vidaletti Ruas, Fernando C. Wehrmeister, Aluisio J. D. Barros

**Affiliations:** 1 Centro Internacional de Equidade em Saúde, Universidade Federal de Pelotas, Pelotas, Brasil.; 2 Escola de Saúde Pública, Universidade de São Paulo, São Paulo, Brasil.

**Keywords:** Health Information Systems, Live Births, Health Status Disparities, Scoping Review, Sistemas de Informação em Saúde, Nascidos Vivos, Desigualdades em Saúde, Revisão de Escopo, Sistemas de Información en Salud, Nacidos Vivos, Desigualdades en la Salud, Revisión de Alcance

## Abstract

The Brazilian Information System on Live Births (SINASC) is widely used by health managers to plan and monitor maternal and child health programs. Scientific research using SINASC data is also essential to identify underexplored topics that warrant further investigation. This study aims to describe the profile of scientific output based on SINASC data and to determine gaps in the literature, with particular attention to studies addressing health inequalities. A scoping review was conducted, including original articles reporting primary outcomes derived from SINASC data. Five databases were searched up to August 2025: PubMed, Embase, Scopus, Web of Science, and LILACS. Brazilian official websites were also manually consulted. A total of 7,363 records were identified, and 85 studies were included in the final analysis. The most frequently analyzed variables were congenital anomalies, birth weight, pregnancy duration, type of delivery, and maternal age. The most commonly reported outcomes were cleft lip/palate, prematurity, low birth weight, Cesarean section, and maternal age distribution. Almost all studies (95.3%) addressed at least one inequality dimension, most frequently geographic and maternal sociodemographic factors. The Human Development Index was the main external socioeconomic indicator. This review underscores the relevance of SINASC for advancing research on health equity in Brazil. Nonetheless, gaps remain in analyses of antenatal care and race/skin color.

## Abstract (pt)

O Sistema de Informações sobre Nascidos Vivos (SINASC) é amplamente utilizado para apoiar gestores de saúde no planejamento e monitoramento de programas de saúde materna e infantil. Pesquisas científicas usando dados do SINASC também são essenciais para identificar temas ainda não explorados que merecem investigação adicional. Este estudo tem como objetivo descrever o perfil da produção científica com base nos dados do SINASC, identificando lacunas na literatura existente, com atenção especial aos estudos que abordam desigualdades em saúde. Foi realizada uma revisão de escopo, incluindo artigos originais que relatavam os resultados primários do SINASC. Cinco base de dados foram pesquisadas até agosto de 2025: PubMed, Embase, Scopus, Web of Science e LILACS. Os sites oficiais brasileiros também foram consultados manualmente. Um total de 7.363 registros foi identificado, e 85 foram incluídos na análise final. As variáveis mais frequentemente analisadas foram anomalias congênitas, peso ao nascer, duração da gestação, tipo de parto e idade materna, com desfechos mais frequentemente relacionados à fenda labial/palatina, prematuridade, baixo peso ao nascer, cesariana e distribuição etária materna. Quase todos os estudos (95,3%) abordaram pelo menos uma dimensão da desigualdade, sendo mais frequentemente fatores geográficos e sociodemográficos maternos, com o Índice de Desenvolvimento Humano como principal variável socioeconômica externa. Esta revisão ressalta a relevância do SINASC para o avanço da pesquisa sobre equidade em saúde no Brasil. No entanto, persistem lacunas nas análises do cuidado pré-natal e da raça/cor da pele.

## Abstract (es)

El Sistema de Informaciones sobre Nacidos Vivos (SINASC) de Brasil se utiliza ampliamente para ayudar a los gestores sanitarios en la planificación y el seguimiento de los programas de salud materno-infantil. La investigación científica que utiliza datos de SINASC también es esencial para identificar temas aún no explorados que merecen una investigación más profunda. Este estudio tiene como objetivo describir el perfil de la producción científica con base en los datos del SINASC, identificando las lagunas en la literatura existente, con especial atención a los estudios que abordan las desigualdades en salud. Se realizó una revisión de alcance, que incluyó artículos originales que informaron sobre los resultados principales del SINASC. Se realizaron búsquedas en cinco bases de datos hasta agosto del 2025: PubMed, Embase, Scopus, Web of Science y LILACS. También se consultaron manualmente los sitios *web* oficiales brasileños. Se identificó un total de 7.363 registros, de los cuales 85 se incluyeron en el análisis final. Las variables analizadas con mayor frecuencia fueron las anomalías congénitas, el peso al nacer, la edad gestacional, el tipo de parto y la edad materna, mientras que los resultados más frecuentemente relacionados fueron el labio hendido/paladar hendido, la prematuridad, el bajo peso al nacer, la cesárea y la distribución de la edad materna. Casi todos los estudios (95,3%) abordaron al menos una dimensión de la desigualdad, centrándose con mayor frecuencia en factores geográficos y sociodemográficos maternos, y el Índice de Desarrollo Humano fue la principal variable socioeconómica externa. Esta revisión destaca la relevancia del SINASC para el avance de la investigación sobre equidad en salud en Brasil. Sin embargo, persisten lagunas en los análisis sobre la atención prenatal y la raza/color de piel.

## Introduction

Health information systems (HIS) are fundamental tools for public health decision-making, supporting the surveillance of health conditions and the evaluation of healthcare system performance ^1^. In this context, it is essential to distinguish between their primary use - focused on administrative management, financial allocation, and routine surveillance - and their secondary use, which involves analyzing existing databases to address research questions and generate scientific knowledge. HIS can provide essential data for monitoring health disparities, facilitating the identification of vulnerable areas and populations and informing public policies and interventions over time ^2^.

The Brazilian Information System on Live Births (SINASC, acronym in Portuguese) is one of the most important and widely used HIS in Brazil. Established in 1990 by the Brazilian Ministry of Health, its primary objective is to collect data on live births nationwide ^3^. It is extensively used by researchers for epidemiological studies and by health managers to plan, monitor, and evaluate maternal and child health programs. The system is based on the Live Birth Certificate (in Portuguese: *Declaração de Nascido Vivo* - DNV), which serves as its main data source ^4^.

SINASC is crucial for assessing epidemiological profiles and monitoring antenatal care, pregnancy, and childbirth ^5^. By providing a comprehensive overview of live births, the system is central to evaluating maternal and child health, access to and quality of care, and disparities in key outcomes. Although coverage increased from 92.9% in 2000 to 98.2% in 2020 ^5^, the system still faces significant challenges. These include variability in data completeness across municipalities and concerns about the quality of specific variables, such as congenital anomalies and race/skin color, which may be underreported or misclassified. Furthermore, changes to the DNV over time require methodological caution when analyzing temporal trends ^5^.

Despite these limitations, SINASC remains an essential tool for research. Its high national coverage and population-based framework provide unique large-scale insights into birth outcomes. Given its significance, understanding the scope of scientific research using SINASC data is imperative not only for identifying underexplored topics but also for examining how researchers have addressed data quality issues. While the body of evidence has expanded, the most recent study analyzing the types of research conducted and the knowledge gaps based on SINASC data was published in 2011 ^6^. Therefore, this study aims to describe the profile of scientific output based on SINASC data and to identify gaps in the literature, with particular attention to studies addressing health inequalities.

## Methods

A scoping review of the literature was conducted and reported in accordance with the *Preferred Reporting Items for Systematic Reviews and Meta-Analyses for Scoping Reviews* (PRISMA-ScR) ^7^.

### Eligibility criteria

Original articles published in peer-reviewed journals that included primary outcomes derived from the SINASC database were eligible. The scope was restricted to studies with a primary analytical focus on Brazil as a whole or on one or more of its five macroregions. Studies limited to a single Federative Unit, municipality, or geographic area smaller than a macroregion (e.g., mesoregion or health microregion) were excluded. However, national or regional studies that included sub-analyses at the state or municipal level were maintained. Studies were excluded if SINASC data were used solely as a denominator to calculate outcomes based on other data sources (e.g., neonatal mortality rates, for which the number of live births from SINASC is typically used as the denominator) or if they focused exclusively on assessing SINASC data coverage, completeness, validity, reliability, or quality. Multicountry studies without a specific focus on Brazil, as well as preprints and gray literature (such as theses and dissertations) were also excluded. No restrictions were applied regarding year of publication or language.

### Information sources and search strategy

Searches were conducted in PubMed (MEDLINE), Embase (Elsevier), Scopus (Elsevier), Web of Science (Clarivate), and the Virtual Health Library (LILACS) up to August 2025. The search strategy was developed in collaboration with a research librarian. Search terms were organized into two blocks. The first included the keywords “Health Information Systems”, “Live Birth Information System”, “Live Births Information System”, “Live Birth System”, “Certificate of Live Birth”, SINASC, DATASUS, and TABNET, combined using the Boolean operator OR. The second block included terms to restrict the search to the Brazilian context and specifically to SINASC. The two blocks were then combined using the AND operator. Portuguese equivalents of the key terms were included in the LILACS search. All articles were managed using Rayyan (https://www.rayyan.ai/) ^8^. Additionally, a manual search was conducted on official websites of Brazilian data observatories, such as the Center for Data and Knowledge Integration for Health (CIDACS; https://cidacs.bahia.fiocruz.br/). The full search strategy is presented in Supplementary Material (https://cadernos.ensp.fiocruz.br/static//arquivo/suppl-e00197425_1782.pdf).

### Selection process

Two reviewers (N.L. and F.S.C.) independently screened the articles according to the predefined eligibility criteria. Titles and abstracts were assessed first, followed by full-text review. Disagreements were resolved by consensus; when necessary, a third reviewer (L.E.S.S.) was consulted.

### Data charting process and items

Following study selection, information from each selected study was independently extracted by two reviewers (N.L. and F.S.C.) and recorded in a Microsoft Excel spreadsheet (https://products.office.com/). Any discrepancies were discussed and resolved by consensus, with consultation with a third reviewer (L.E.S.S.) when needed. The extracted data are available in Supplementary Material (https://cadernos.ensp.fiocruz.br/static//arquivo/suppl-e00197425_1782.pdf).

Given that all included studies used data from SINASC, it is important to briefly describe this information system and its structure. SINASC uses the DNV as a standardized data collection instrument, as established by the Brazilian Ministry of Health. The DNV collects sociodemographic information on the mother and the newborn, including maternal age, education, race/skin color, place of residence, and newborn sex. It also records clinical information related to pregnancy and childbirth, such as gestational age, type of pregnancy, mode of delivery, number of antenatal care visits, birth weight, and presence of congenital anomalies, as well as data on childbirth care, including place of occurrence and type of health professional attending the delivery. These data are essential for maternal and child health surveillance, enabling the characterization of epidemiological profiles, monitoring antenatal and childbirth care, and identifying health inequalities. Operational definitions, response categories, and guidance for proper completion of the DNV are provided in official manuals and technical documents issued by the Brazilian Ministry of Health ^4^ ^,^ ^5^.

Based on this structure, the following information was extracted from each article: study identification (author, year of publication, and title), objective, setting (Brazil or its regions), study period, SINASC variables used to calculate the outcomes, outcomes assessed, primary exposures or stratification variables, assessment of interaction or double stratification, primary analyses conducted (trend analysis, geospatial analysis, descriptive analysis, or multivariable analysis), unity of analysis (ecological and/or individual), level of geographical aggregation, and main results. Geographic region, maternal age, race/skin color, marital status, and education were among the exposure or stratification variables collected from the original studies. In addition to the variables available in the SINASC dataset, we also extracted information on external socioeconomic indicators used in the studies included in this scoping review.

The classification of the unit of analysis as individual (i.e., each live birth in SINASC) or ecological (e.g., municipalities) was based on the level of data aggregation. Ecological analyses involve aggregating data at the group or population level, such as municipalities, states, or meso- and macroregions. Descriptive analyses were classified as individual because, although they present aggregated results, they are conducted at the individual level.

Additionally, to assess the impact of the publications, we collected data in September 2025 on the journal in which each article was published, its journal impact factor (JIF), and its JIF category ranking, as reported in the 2024 *Journal Citation Reports* ^9^. Category rankings are based on the journal’s position within its subject area according to JIF, from which percentile and quartile classifications are derived. We also obtained the number of documents citing each publication from Scopus. Given the Brazilian context of this study, the included articles were evaluated according to the current Qualis-CAPES ranking (2021-2024 quadrennium). The Qualis system assesses the quality of scientific production by classifying the journals in which articles are published, a metric used to evaluate Brazilian graduate programs. Journals are categorized as A1 to A4 (highest), B1 to B4, and C (lowest). The classification for each journal was retrieved from the Qualis Periódicos portal, available at: https://sucupira-legado.capes.gov.br/sucupira/public/consultas/coleta/veiculoPublicacaoQualis/listaConsultaGeralPeriodicos.jsf.

### Synthesis of results

Data were analyzed quantitatively, with absolute and relative frequencies reported for the overall sample and stratified by period: before 2021 and from 2021 onward. The year 2021 was considered a milestone for evaluating contemporary literature, as it captures scientific production during the COVID-19 pandemic. Additionally, this timeframe corresponds to the most recent updates to the DNV terminology, including the replacement of the fields “mother’s name” and “father’s name” with “parturient” and “legal guardian” ^4^ ^,^ ^5^, reflecting the current standardization of the system’s data collection procedures.

## Results

**Figure 1:**
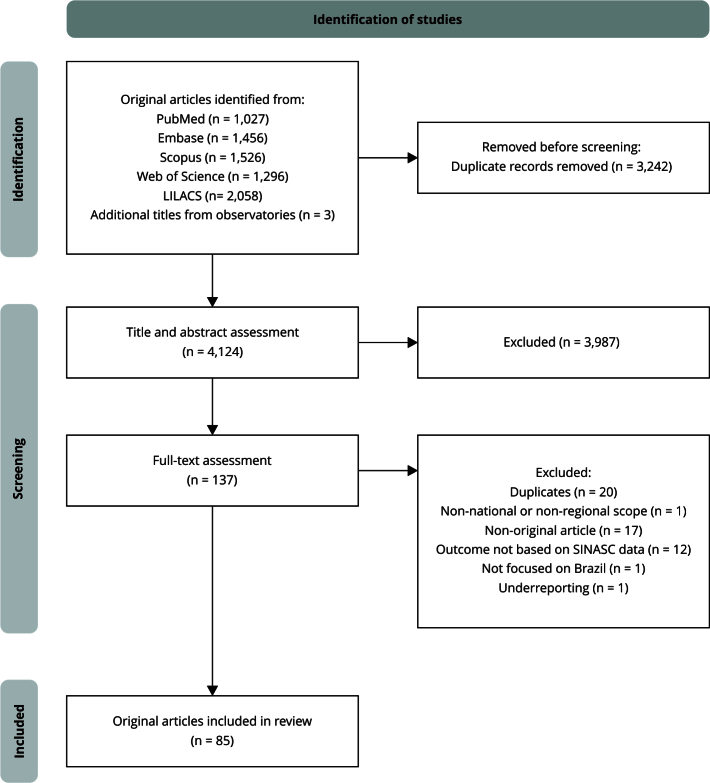
Flow diagram of the scoping review.

A total of 7,363 publications were initially identified, including three additional articles retrieved from observatory websites. After removing duplicates, 4,124 studies remained. Following title and abstract screening, 137 publications were selected for full-text review. After a detailed assessment, 85 studies were included in the final analysis (Figure 1). Most studies were published in the last five years (n = 49, 57.6%) and had a national scope (n = 81, 95.3%). The remaining studies focused on a single region: the South (n = 3, 3.5%) and the Northeast (n = 1, 1.2%).

SINASC: Brazilian Information System on Live Births.

Table 1 summarizes the main findings. The most frequently used SINASC variables were congenital anomaly (n = 34; 40%) ^10^ ^,^ ^11^ ^,^ ^12^ ^,^ ^13^ ^,^ ^14^ ^,^ ^15^ ^,^ ^16^ ^,^ ^17^ ^,^ ^18^ ^,^ ^19^ ^,^ ^20^ ^,^ ^21^ ^,^ ^22^ ^,^ ^23^ ^,^ ^24^ ^,^ ^25^ ^,^ ^26^ ^,^ ^27^ ^,^ ^28^ ^,^ ^29^ ^,^ ^30^ ^,^ ^31^ ^,^ ^32^ ^,^ ^33^ ^,^ ^34^ ^,^ ^35^ ^,^ ^36^ ^,^ ^37^ ^,^ ^38^ ^,^ ^39^ ^,^ ^40^ ^,^ ^41^ ^,^ ^42^ ^,^ ^43^, birth weight (n = 19; 19.5%) ^17^ ^,^ ^28^ ^,^ ^36^ ^,^ ^44^ ^,^ ^45^ ^,^ ^46^ ^,^ ^47^ ^,^ ^48^ ^,^ ^49^ ^,^ ^50^ ^,^ ^51^ ^,^ ^52^ ^,^ ^53^ ^,^ ^54^ ^,^ ^55^ ^,^ ^56^ ^,^ ^57^ ^,^ ^58^ ^,^ ^59^, pregnancy duration (n = 18; 21.2%) ^17^ ^,^ ^28^ ^,^ ^36^ ^,^ ^44^ ^,^ ^46^ ^,^ ^49^ ^,^ ^51^ ^,^ ^52^ ^,^ ^55^ ^,^ ^56^ ^,^ ^59^ ^,^ ^60^ ^,^ ^61^ ^,^ ^62^ ^,^ ^63^ ^,^ ^64^ ^,^ ^65^ ^,^ ^66^, type of delivery (n = 15; 17.6%) ^36^ ^,^ ^56^ ^,^ ^67^ ^,^ ^68^ ^,^ ^69^ ^,^ ^70^ ^,^ ^71^ ^,^ ^72^ ^,^ ^73^ ^,^ ^74^ ^,^ ^75^ ^,^ ^76^ ^,^ ^77^ ^,^ ^78^ ^,^ ^79^, and maternal age (n = 12; 14.1%) ^51^ ^,^ ^56^ ^,^ ^69^ ^,^ ^78^ ^,^ ^80^ ^,^ ^81^ ^,^ ^82^ ^,^ ^83^ ^,^ ^84^ ^,^ ^85^ ^,^ ^86^ ^,^ ^87^. Antenatal care visits were investigated in eight publications ^17^ ^,^ ^56^ ^,^ ^69^ ^,^ ^78^ ^,^ ^88^ ^,^ ^89^ ^,^ ^90^ ^,^ ^91^, Apgar scores in six ^17^ ^,^ ^36^ ^,^ ^55^ ^,^ ^92^ ^,^ ^93^ ^,^ ^94^, and type of pregnancy in only two studies ^36^ ^,^ ^95^. Although the ranking of these variables varied slightly across time periods, they consistently remained among the most frequently examined, both before and after 2021. The most commonly investigated outcomes associated with these variables were cleft lip and/or palate, prematurity, low birth weight, Cesarean section, and distribution of live births by maternal age.

**Table 1:** Descriptive analysis of the publications evaluated.

	Total		Before 2021		2021 or after	
	n	%	n	%	n	%
Outcomes (SINASC variables)						
Antenatal care visits	8	9.4	4	11.1	4	8.2
Apgar	6	7.1	3	8.3	3	6.1
Birth weight	19	22.4	9	25.0	10	20.4
Maternal age	12	14.1	6	16.7	6	12.2
Pregnancy duration	18	21.2	7	19.4	11	22.4
Congenital anomaly	34	40.0	11	30.6	23	46.9
Type of delivery	15	17.6	10	27.8	5	10.2
Type of pregnancy	2	2.4	2	5.6	0	0.0
Others *	2	2.4	2	5.6	0	0.0
Primary exposures or stratification variables						
Double stratification/interaction	22	25.9	9	25.0	13	26.5
Socioeconomic and demographic factors from SINASC						
Geographical	65	76.5	29	80.6	36	73.5
Maternal age	29	34.1	11	30.6	18	36.7
Maternal race/skin-color	22	25.9	7	19.4	15	30.6
Maternal marital status	24	28.2	11	30.6	13	26.5
Maternal education	42	49.4	15	41.7	27	55.1
External socioeconomic factors						
HDI	10	11.8	7	19.4	3	6.1
GDP per capita	2	2.4	1	2.8	1	2.0
Household income per capita	4	4.7	3	8.3	1	2.0
Illiteracy	2	2.4	2	5.6	0	0.0
Others **	15	17.6	10	27.8	5	10.2
Primary analysis						
Trend or change	56	65.9	24	66.7	32	65.3
Geospatial	6	7.1	2	5.6	4	8.2
Descriptive	52	61.2	23	63.9	29	59.2
Multivariable	23	27.1	6	16.7	17	34.7
Unity of analysis						
Ecological	26	30.6	15	41.7	11	22.4
Individual	28	32.9	7	19.4	21	42.9
Ecological/individual	31	36.5	14	38.9	17	34.7
Geographical disaggregation level						
National	49	86.0	24	82.8	25	89.3
Regional	41	71.9	19	65.5	22	78.6
State	15	26.3	11	37.9	4	14.3
Municipal	5	8.8	2	6.9	3	10.7
Microregion	1	1.8	1	3.4	0	0.0
Health districts	1	1.8	1	3.4	0	0.0
Type of publication						
National	50	58.8	25	69.4	25	51.0
International	35	41.2	11	30.6	24	49.0
JIF quartile						
Q1	13	15.3	5	13.9	8	16.3
Q2	15	17.6	7	19.4	8	16.3
Q3	29	34.1	18	50.0	11	22.4
Q4	11	12.9	3	8.3	8	16.3
No classification	17	20.0	6	16.7	11	22.4
Qualis-CAPES classification						
A1 (highest quality level)	30	35.3	10	27.8	20	40.8
A2	20	23.5	9	25.0	11	22.5
A3	7	8.2	4	11.1	3	6.1
A4	11	12.9	7	19.4	4	8.2
B1	2	2.4	0	0.0	2	4.1
B2	1	1.2	0	0.0	1	2.0
B3	1	1.2	0	0.0	1	2.0
B4	3	3.5	1	2.8	2	4.1
C (lowest quality level)	2	2.4	0	0.0	2	4.1
Not classified	8	9.4	5	13.9	3	6.1

GDP: gross domestic product; HDI: Human Development Index; JIF: journal impact factor; SINASC: Brazilian Information System on Live Births.

Note: a total of 85 studies is represented, with 36 studies published before 2021 and 49 in 2021 or subsequent years. The variables Outcomes (SINASC variables), Primary exposures or stratification variables, Primary analysis, and Geographical disaggregation level do not sum up 100% in the column, since a study may be classified into more than one category.

* Maternal education, marital status, race/skin color, fetal presentation, labor onset - each evaluated in one study;

** Area of residence, household conditions, maternal race/skin color, income per capita, Gini index, average years of schooling, percentage of population with income less than half a minimum wage per capita, percentage of households with sewage, percentage of households with garbage collection, adult population with low income - each evaluated in one study.

The exposure or stratification variables included socioeconomic and demographic factors from SINASC and socioeconomic data from external sources. A total of 81 studies (95.3%) evaluated at least one dimension of inequality, and 22 (25.9%) examined interactions or performed double stratification. Geographic area was the most frequently assessed dimension (n = 65, 76.5%), with regions being the most commonly analyzed units. Other frequent factors included maternal sociodemographic characteristics, particularly education (n = 42, 49.4%), age (n = 29, 34.1%), marital status (n = 24, 28.2%), and race/skin color (n = 22, 25.9%). A smaller number of studies used external data from SINASC. The Human Development Index (HDI) was the most frequently used variable obtained from external sources, appearing in ten studies (11.8%), followed by household income per capita (n = 4, 4.7%), gross domestic product (GDP) per capita at the municipal level (n = 2, 2.4%), and illiteracy (n = 2, 2.4%), among others. From 2021 onwards, HDI, GDP per capita, and illiteracy were used. Overall, external socioeconomic factors were more frequently assessed in studies published before 2021 (Table 1).

Trend or change analysis was the most common analytical approach (n = 56, 65.9%), followed by descriptive analysis (n = 52, 61.2%). Multivariable analysis was conducted in 23 studies (27.1%), and geospatial analysis in six (7.1%). Although the ranking of these approaches remained unchanged over time, the use of multivariable analysis increased from 16.7% before 2021 to 34.7% thereafter (Table 1).

Regarding the unit of analysis, when combining studies using exclusively ecological approaches (n = 26, 30.6%) with those using both ecological and individual levels (n = 31, 36.5%), ecological-level analyses were conducted in 57 studies (67.1%). Similarly, individual-level analyses, whether used alone (n = 28, 32.9%) or in combination, were performed in 59 studies (69.4%). From 2021 onwards, a shift was observed: individual-level analyses (alone or combined) were present in 38 studies (77.6%), compared to 21 studies (58.3%) published before 2021. Among ecological studies, the national level was the most frequent unit of geographic analysis (n = 49, 86%), followed by regional (n = 41, 71.9%) and state-level (n = 15, 26.3%) analyses. Percentages exceeded 100% because several studies examined multiple geographical levels. Municipal-level analyses were less common (n = 5, 8.8%), and microregional and health district-level analyses were the least frequent, appearing in only one study. The distribution of geographic disaggregation levels was similar across periods, with national and regional levels remaining the primary focus (Table 1).

There was a predominance of articles published in Brazilian journals, accounting for 58.8% (n = 50) of the total, while international journals represented 41.2% (n = 35). Among studies published from 2021 onwards, 49% appeared in international journals, compared to 30.6% before 2021. Regarding impact factors, 68 articles were published in journals with an available JIF classification, most of which were in the third and fourth quartiles (n = 40, 58.8%). Among studies with information available in the most recent JIF classification, 22.8% of those published in the last five years appeared in top-quartile journals (Q1), compared to 15.2% before 2021 (Table 1). At the time of data extraction, citation counts ranged from 0 to 67. Nine studies lacked citation information. The three most cited articles addressed microcephaly in Brazil, the Zika virus epidemic, and Cesarean section rates, respectively. Overall, 35.3% of the studies were published in journals ranked in the highest category (A1) of the Qualis-CAPES system.

## Discussion

This scoping review provides a comprehensive overview of the scientific literature using SINASC data for analyses at the national or regional level, focusing on how health inequalities have been addressed. The findings reveal a growing body of research that increasingly relies on SINASC to explore diverse maternal and neonatal health outcomes.

Our analysis indicates that most studies included in this review were published in the last five years and had national coverage. This trend likely reflects the increasing accessibility and use of SINASC data, which now covers nearly 90% of Brazilian municipalities ^96^ ^,^ ^97^ and shows high completeness of variables ^98^. These advances have established the system as the main official source of information on live births in Brazil, with strong potential to support large-scale epidemiological investigations.

The most frequently analyzed outcomes included congenital anomalies, prematurity, low birth weight, Cesarean section, and distribution of live births by maternal age. Antenatal care, particularly the number of visits and timely initiation, was notably underexplored. Given its importance in preventing adverse outcomes ^99^ ^,^ ^100^, further investigation into antenatal care practices is warranted, especially regarding disparities in access. Future studies should leverage SINASC data to address these gaps in maternal healthcare.

Regarding the analysis of inequalities, our findings emphasize a strong focus on geographic disparities, followed by maternal education, age, marital status, and race/skin color. Notably, maternal race/skin color remains underexplored, despite being a key social determinant associated with substantial inequalities in maternal and child health outcomes in Brazil ^101^ ^,^ ^102^ ^,^ ^103^ ^,^ ^104^ ^,^ ^105^, where structural racial disparities persist. Although over 90% of the studies assessed at least one dimension of inequality, only about one third explored intersections between different dimensions, suggesting an opportunity for more nuanced analytical approaches. Greater integration of an intersectional perspective is warranted to better understand how interacting social factors compound their effects on health outcomes.

External socioeconomic variables, such as HDI and GDP per capita, were more prevalent in studies published before 2021, suggesting a decline in the incorporation of broader contextual data in recent years. This shift aligns with the observed increase in studies employing individual-level analyses. Nevertheless, the inclusion of contextual socioeconomic factors remains essential to understand the structural determinants of health disparities. In Brazil, several contextual indicators can support future research on the broader social determinants of health. For example, the Brazilian Deprivation Index (IBP, acronym in Portuguese) ^18^ and the Social Vulnerability Index (IVS, acronym in Portuguese) ^19^ capture territorial inequalities by providing standardized measures of deprivation and vulnerability at the municipal level. These indices enable more detailed analyses and can inform the development of geographically targeted and socially responsive public health policies.

Most of the identified studies focused on national or regional results. However, within these scopes, stratification was predominantly limited to macroregions. While such analyses help identify large-scale patterns, they often overlook essential variations at finer levels of granularity. Analyses at the state, municipal, and microregional levels remain underutilized as sub-analyses within broader investigations, despite the availability of disaggregated SINASC data. This gap represents a missed opportunity to uncover localized health inequities and to inform more context-sensitive public health interventions. Future studies should prioritize deeper geographic disaggregation to better support targeted policy responses. It is important to note that this review focused exclusively on national and regional research; consequently, studies conducted at the state or municipal level were not included. Although expanding the search to encompass strictly local studies could generate valuable evidence for health management, our findings highlight that even large-scale research often fails to explore the geographic disaggregation potential offered by the system.

Regarding scientific impact, the increasing number of articles published in international and higher-impact journals since 2021 suggests growing global recognition of Brazil’s live birth data and the expanding relevance of SINASC-based research. A key strength of this scoping review is its systematic and comprehensive approach, including a broad search strategy across five major databases and adherence to the PRISMA-ScR guidelines. The inclusion of studies regardless of language or year of publication further enhances the completeness of our synthesis. However, some limitations should be acknowledged. Despite efforts to ensure comprehensive coverage, relevant publications may have been missed, particularly those not indexed in the selected databases or published in non-traditional formats. Moreover, some studies may have been overlooked because they did not explicitly mention SINASC in indexed fields, potentially limiting the scope of inclusion. Finally, our focus on national and regional studies may have excluded valuable local-level research that could offer context-specific insights into health disparities.

Overall, this review demonstrates the extensive use of SINASC data in the scientific literature and its growing potential to advance research on health equity in Brazil. However, important gaps remain. The limited focus on antenatal care, the relatively infrequent use of race/skin color as a primary exposure or stratification variable, and the underutilization of local-level analyses reveal critical areas for future investigation. Incorporating indicators from complementary data sources may enhance analyses of the structural and social determinants underlying health inequalities. Addressing these gaps is essential to improve understanding of health disparities and to inform more equitable, evidence-based maternal and neonatal health policies in Brazil.

## Data Availability

The sources of information used in the study are indicated in the body of the article.
