# Supplementary material for: Scientific production on health inequalities using the Brazilian Information System on Live Births: a scoping review
Source: Cad Saude Publica. 2026 Jul 31;42:e00197425. doi: 10.1590/0102-311XEN197425 (PMC13431994; doi:10.1590/0102-311XEN197425)
Supplement: Supplementary Material [file 1678-4464-csp-42-EN197425-s.pdf]

Supplementary Table S1. Detailed search strategy for the scoping review

Database	Search strategy
**PubMed**<br>All fields	((((((((("Health Information Systems") OR ("Live Birth Information System")) OR ("Live Births Information System")) OR ("Live Birth System")) OR ("Certificate of Live Birth")) OR (SINASC)) OR (DATASUS)) OR (TABNET)) AND (Brazil*)
**Embase**<br>Title, abstract, and keywords	('health information systems':ti,ab,kw OR 'live birth information system':ti,ab,kw OR 'live births information system':ti,ab,kw OR 'live birth system':ti,ab,kw OR 'certificate of live birth':ti,ab,kw OR sinasc:ti,ab,kw OR datasus:ti,ab,kw OR tabnet:ti,ab,kw) AND brazil*:ti,ab,kw
**Scopus**<br>Title, abstract, and keywords	(TITLE-ABS-KEY (("Health Information Systems" OR "Live Birth Information System" OR "Live Births Information System" OR "Live Birth System" OR "Certificate of Live Birth" OR SINASC OR DATASUS OR TABNET)) AND TITLE-ABS-KEY ((Brazil*)))
**Web of Science**<br>All fields	(ALL=("Health Information Systems") OR ALL=("Live Birth Information System") OR ALL=("Live Births Information System") OR ALL=("Live Birth System") OR ALL=("Certificate of Live Birth") OR ALL=(SINASC) OR ALL=(DATASUS) OR ALL=(TABNET)) AND ALL=("Brazil*")
**LILACS**<br>Title, abstract, and subject	("Sistemas de Informação em Saúde" OR "Sistema de Informação sobre Nascidos Vivos" OR "Sistema de Informações sobre Nascidos Vivos" OR "Health Information Systems" OR "Live Birth Information System" OR "Live Births Information System" OR "Live Birth System" OR "Certificate of Live Birth" OR SINASC OR DATASUS OR TABNET) AND (Brasil* OR Brazil*)

Supplementary Table S2. Detailed description of the publications included in the scoping review

Author, publication year	Study objective	Setting	Study period	Outcomes (SINASC variables)	Primary exposures and stratification variables
**Wehrmeister et al., 2025**	To provide a simple guide to analyses of health inequalities, focusing on women's and children's health-related outcomes. Throughout the article, we present analyses as practical examples to demonstrate how to assess and interpret health disparities.	Brazil	2020-2022	Antenatal care visits	Region, maternal education
**Souza et al., 2025**	To identify areas at risk for congenital anomalies in Brazil, from 2012 to 2021	Brazil	2012-2021	Congenital anomaly	Moran's global index, region
**Cruz et al., 2025**	To assess the prevalence of congenital ophthalmic malformations in Brazil between 2013 and 2021and study the factors related to their occurrence in live births during this period.	Brazil	2013-2021	Congenital anomaly	Antenatal care, type of delivery, type of pregnancy, maternal education, gestational age, apgar, skin color/race, maternal age, sex and birthweight of the child
**Fernandes et al., 2025**	This study investigated the socioeconomic and biological factors associated with congenital anomalies in Brazil.	Brazil	2012-2020	Congenital anomaly	Maternal education, maternal race/skin color, region maternal marital status, pregnancy month that started prenatal appointment, maternal age in years, number of previous fetal losses, and type of pregnancy
**Santos et al., 2025**	To identify spatial clustering and maternal and birth-related factors associated with the incidence of orofacial clefts in Brazil from 2001 to 2022	Brazil	2001-2023	Congenital anomaly	Region, maternal age, maternal education, race/ethnicity, preterm birth, and birthweight
**Sá da Silva et al., 2025**	To investigate the timely initiation of antenatal care among Brazilian adolescents to support the national discussion on the gestational age limit for legal abortion.	Brazil	2020-2022	Antenatal care visits	Region, maternal age, race/skin color, and education.
**Melkan et al., 2025**	To identify potential associations of this prevalence with socioeco - nomic, gestational, and regional factors by performing an analysis using data sourced from the Live Birth Information System (*Sistema de Informações sobre os Nascidos Vivos* – SINASC) covering the period from 2011 to 2020	Brazil	2011-2020	Congenital anomaly	Region, ethnicity/color, level of education, marital status, number of prenatal visits, type of pregnancy, gestational age, type of delivery, and sex of the child.
**Gomes et al., 2025**	To describe the prevalence of and main outcomes related to these anomalies in Brazil.	Brazil	2010-2022	Congenital anomaly	Region, maternal age, maternal education, self-declared maternal race, number of prenatal consultations, number of fetal losses or previous miscarriages, and type of pregnancy.
**Vieira et al., 2025**	To analyze distribution of orofacial cleft cases (cleft palate, cleft lip and cleft palate with cleft lip) and their temporal trend in Brazil, according to the country's regions and Federative Units from 2010 to 2021, in addition to comparing proportions during the COVID-19	Brazil	2010-2021	Congenital anomaly	Brazilian regions and Federative Units

	pandemic, from 2020 to 2021, with the preceding time series, from 2010 to 2019.				
**Rocha et al., 2025**	To evaluate the association between interpregnancy weight change using different metrics (changes in BMI units, changes in BMI category, and percentage weight change) and adverse birth outcomes (preterm birth, Low Birth Weight, and macrosomia) in the sub-sequent pregnancy using administrative-linked data from Brazil.	Brazil	2008-2015	Preterm birth, low birth weight(LBW) and macrosomia	Region of residence, residence area, household overcrowding, maternal education, marital status, maternal race/ethnicity, maternal age at delivery, number of prenatal visits, type of delivery, pre-pregnancy BMI, interbirth interval, and interpregnancy weight change.
**Cerqueira-Silva et al., 2025**	To evaluate the characteristics of women giving birth who are internal migrants in Brazil, to investigate whether offspring of internal migrant women in Brazil have poorer perinatal outcomes compared to non-migrant Brazilians and to investigate whether the risk of poor perinatal outcomes in offspring of internal migrant women differs based on their migration to municipalities with equal/higher or lower Human Development Index (HDI) than their original municipalities.	Brazil	2011-2018	Timely initiation of antenatal care, preterm birth, birthweight, gestational age, Apgar score, congenital anomaly at birth	Internal migrant, municipality of registration, and the municipality of birth Human Development Index (HDI), age of the mother, state of residence, number of antenatal visits, education level, year of conception, number of previous pregnancies (0 or ≥1), receipt of conditional cash transfer benefit, marital status, and previous fetal loss.
**Rebouças et al., 2025**	To quantify the magnitude of ethnic-racial inequalities in adverse birth and neonatal outcomes in a cohort of more than 23 million Brazilian newborns	Brazil	2012-2019	Premature birth, birthweight	Maternal race/skin color, Maternal education, age, parity, and type of delivery
**Cargnin et al., 2024**	To analyze the temporal trend of live births by Brazilian regions and states, considering the reproductive age during pregnancy.	Brazil	2011-2021	Maternal age	Region, state
**da Silva et al., 2024**	We aimed to estimate the association between syphilis in pregnancy and adverse outcomes at birth using a national population-based database. Additionally, we investigated the association according to non-treponemal titer and maternal treatment status and role of prenatal care.	Brazil	2011-2017	Birthweight, pregnancy duration	Gestational syphilis, VDRL titer, gestational syphilis treatment
**de Macedo filho et al., 2024**	We aim to assess the prevalence, surgical intervention, hospitalization costs associated with shunt procedures, and mortality in congenital hydrocephalus compared with pediatric acquired hydrocephalus using DATASUS, a Brazilian National Database.	Brazil	2008-2021	Congenital anomaly	None
**Moura et al., 2024**	The objective of this study was to analyze the prevalence of CAUL in Brazil from 2010 to 2019, utilizing the DATASUS database, and to evaluate the associated maternal and neonatal variables.	Brazil	2010-2019	Congenital anomaly	Region, maternal age, type of delivery, number of antenatal care visits, maternal education, pregnancy duration, type of pregnancy, birthweight, sex, newborn's race/skin

					color, Apgar at 1 and 5 minutes
**Ortelan et al., 2024**	We investigated the association between receiving BF assistance throughout pregnancy and the occurrence of PTB, considering both overall and their severity levels (moderate-to-late, severe, and extreme). Additionally, we aimed to explore whether the observed association varies based on prenatal care and the quality of BF management in the mothers' municipalities.	Brazil	2012-2015	Pregnancy duration	Bolsa Família
**Pinto et al., 2024**	This study aimed to analyze the spatial distribution of pregnancy in children under 14 years and six months by Brazilian region and municipality from 2011 to 2021 and the sociodemographic and health characteristics of parturients and live births during pregnancy, delivery, and birth.	Brazil	2011-2021	Maternal age	Region, state, municipality, maternal race/skin color, maternal education, maternal marital status, previous pregnancies, number of live births, type of pregnancy, type of delivery, number of antenatal care visits, pregnancy duration, month antenatal care started, birthweight, Apgar at 5 minute
**Polay et al., 2024**	The objective of this study was to examine epidemiological trends, place of residence, duration of gestation, sex, and race of the newborn diagnosed with congenital glaucoma in Brazil.	Brazil	2017-2021	Congenital anomaly	State, sex, newborn's race/skin color, pregnancy duration
**Silva et al., 2024**	The present study aimed to describe the epidemiological profile and prevalence of live births with isolated orofacial cleft in Brazil between 1999 and 2020.	Brazil	1999-2020	Congenital anomaly	Region, state, sex, newborn's race/skin color, maternal age, paternal age, Apgar at 1 and 5 minutes, previous pregnancies, number of antenatal care visits, place of birth, type of delivery, birthweight, pregnancy duration, type of pregnancy, maternal education, maternal marital status
**Victor et al., 2024**	This study aimed to analyze the temporal trend of fetal growth in newborns in Brazil between 2010 and 2020	Brazil	2010-2020	Birthweight	Region, maternal age
**Zhang et al., 2024**	In this study, we aim to characterize the exposure-time-response (E-T-R) relationship between wildfire-specific PM2.5 and PTB risk, identify the effect modifiers, specify the critical exposure windows, and quantify the spatiotemporal burden, leveraging a large nationwide population-based cohort in Brazil, for up to 10 years.	Brazil	2010-2019	Pregnancy duration	Daily wildfire-specific PM2.5
**Coutinho and Souza, 2024**	The objective of the study was to conduct time-series analyses to explore trends in fertility continuity and changes over the last decade, investigating whether the health and economic crises faced by Brazilian women significantly altered expected trajectories.	Brazil	2010-2022	Maternal age	Region, maternal age, maternal education

**Rocha et al., 2024**	To describe the growth trajectories and to estimate catch-up growth during the first 5 y of life of small newborns according to 3 vulnerability phenotypes (preterm, low –birth weight (LBW) and small-for-gestational age (SGA)).	Brazil	2001-2015	Birthweight	Region, residence area, household overcrowding (> 2 inhabitants per room), maternal race, maternal education, marital status; number of prenatal visits, maternal age at delivery, number of previous pregnancies, type of delivery, and gender of the child
**Alberton et al., 2023**	This study aimed to measure the prevalence of prematurity in Brazil, according to macroregions and maternal characteristics, over the past 11 years, and compare the proportions during the SARS-CoV-2 pandemic, 2020 and 2021, with those of the historical series from 2011 to 2019, pre-pandemic period.	Brazil	2011-2021	Pregnancy duration	Region, maternal age, maternal education, maternal race/skin color, type of pregnancy, number of antenatal care visits, type of delivery
**Brasil et al., 2023**	The present study aims to evaluate environmental factors, such as the place of delivery, type of birth, and number of antenatal visits that may influence changes in the Apgar score, as well as the newborn's weight, as understanding these factors is important for prenatal care	Brazil	1994-2018	Apgar	Place of birth, type of delivery, number of antenatal care visits
**Charles et al., 2023**	In this study, we aim to assess the changes in preterm birth counts in Brazil and its regions, by comparing the number of preterm deliveries during the pandemic (2020 and 2021) and pre-pandemic periods (2017, 2018 and 2019).	Brazil	2017-2021	Pregnancy duration	Region
**da Silva et al., 2023**	The objective of this study was to analyze whether differences in lifestyle and being born in coastal or inland areas are associated with differences in the frequency of OC types.	Brazil	1999-2020	Congenital anomaly	Coast or inland residence, sex, maternal age, newborn's race/skin color, type of pragnancy, number of antenatal care visits, previous pregnancies
**Fernandes et al., 2023**	The present study describes the temporal trend of prevalence and child mortality due to CA in live births (LBs) in Brazil and in geographic regions from 2001 to 2018.	Brazil	2001-2018	Congenital anomaly	Region
**Ferreira et al., 2023**	The study aims to provide comprehensive information, presenting a national overview of childbirth and birth in Brazil, considering different geographic regions.	Brazil	2011-2021	Type of delivery	Region, maternal age, maternal education, maternal marital status, pregnancy duration, type of pregnancy, number of antenatal care visits, Robson classification
**Magalhães et al., 2023**	This study evaluates the predisposing factors of Ap5 < 7 in Brazil as a marker of neonatal prognosis and the change in its indexes in the last twenty years.	Brazil	1999/2018-2019	Apgar	Region, pregnancy duration, birthweight, maternal race/skin color, sex, congenital anomaly, maternal age, maternal education, number of antenatal care visits, number of live births, previous fetal losses or miscarriages, type of pregnancy, type of

					delivery, place of birth
**Oliveira et al., 2023**	The purpose of this study was to verify possible associations of sociodemographic and clinical characteristics in patients with spinal dysraphism.	Brazil	1999-2019	Congenital anomaly	Region, state, sex, newborn's race/skin color, maternal age, maternal education, type of pregnancy, pregnancy duration, birthweight, number of antenatal care visits
**Oliveira et al., 2023**	This paper aims to describe the epidemiology of congenital diaphragmatic hernia (CDH), esophageal atresia (EA), and gastroschisis (GS) in South Brazil (Paraná, Santa Catarina, and Rio Grande do Sul) from 2009 to 2019.	South region	2009-2019	Congenital anomaly	State, maternal age, maternal education, maternal marital status, number of antenatal care visits, type of pregnancy, pregnancy duration, type of delivery, birthweight, sex, newborn's race/skin color, Apgar at 1 and 5 minutes
**Victor et al., 2023**	This study seeks to explore the association between the food environment and small for gestational age (SGA), large for gestational age (LGA) and low birth weight (LBW) newborns.	Brazil	2016	Birthweight, pregnancy duration	Food environment
**Zanon et al., 2023**	This work aims to compare the incidence of spina bifida (SB) before and after folic acid (FA) fortification of baking flours in Brazil.	Brazil	1999-2020	Congenital anomaly	Region
**Dallegrave et al., 2022**	This study aims to perform a descriptive analysis of the occurrence of orofacial clefts in the population of southern Brazil from 2007 to 2016.	South region	2007-2016	Congenital anomaly	State, sex, birthweight, type of delivery, number of antenatal care visits, pregnancy duration, maternal age, maternal education
**Dias et al., 2022**	Our study aimed to describe cesarean section and repeated cesarean section rates in Brazil according to gestational age at birth and type of hospital.	Brazil	2017	Type of delivery	Region, pregnancy duration, type of hospital, maternal age, race/skin color, maternal education, maternal marital status, previous pregnancies
**Lucas et al., 2022**	This study aims to assess the long-term benefits of cash transfers through intergenerational transmission of health and poverty, by assessing the relationship between BF aid received by the mother during childhood and newborn health, controlling for a set of socioeconomic and health variables.	Brazil	2011-2015	Birthweight, congenital anomaly, pregnancy duration	Bolsa Família
**Moura et al., 2022**	To analyze the relationship between vulnerability at birth, represented by the low birth weight variable, and selected variables, such as real GDP per capita and Bolsa Família coverage in the municipality, in addition to characteristics involving maternal age, number of prenatal consultations, race, marital status and years of study.	Brazil	2012-2016	Birthweight	Gross Domestic Product per capita, Bolsa Família coverage, maternal age, number of antenatal care visits, maternal race/skin color, maternal education, maternal marital status
**Bessa et al., 2021**	This study aims to verify possible associations of clinical and sociodemographic characteristics in patients with cryptorchidism.	Brazil	1999-2018	Congenital anomaly	Region, state, maternal age, maternal education, maternal marital status, pregnancy duration, type of pregnancy, place of birth

**Bicalho et al., 2021**	To analyze the temporal trends in the fertility rate, proportion of antenatal consultations and caesarean sections in Brazilian adolescents aged 15 to 19, between 2000 and 2015.	Brazil	2000-2015	Antenatal care visits, maternal age, type of delivery	Region
**Laignier et al., 2021**	This article aimed to estimate the occurrence of DS in Brazil between 2012 and 2018, and to verify its association with the maternal, gestational, and paternal characteristics, and vitality conditions of the newborn.	Brazil	2012-2018	Congenital anomaly	Sex, birthweight, Apgar at 1 and 5 minutes, maternal age, maternal marital status, maternal education, paternal age, number of antenatal care visits, pregnancy duration, type of delivery
**Martinelli et al., 2021**	This study aims to analyze the trend of prematurity in Brazil between 2012 and 2019, according to sociodemographic, prenatal and childbirth characteristics.	Brazil	2012-2019	Pregnancy duration	Region, maternal age, maternal education, maternal race/skin color, number of antenatal care visits, type of delivery
**Melo, 2021**	In this paper, we estimate impacts and unintended consequences of Resolution 2,144 from the Federal council of Medicine (CFM) on outcomes of Low Risk First Born births (LRFB). The Resolution introduces a minimum of 39th weeks of gestation for Elective CS.	Brazil	2015-2017	Type of delivery	None
**Monteiro et al., 2021**	The objectives of this study were to evaluate the frequency of live births (LB) of mothers in the age group of 10–13 years and the age-specific fertility rate (ASFR) in the period 1996–2018.	Brazil	1996-2018	Maternal age	Region
**Paixão et al., 2021**	The objectives of our study were to (1) estimate the caesarean rate in Brazil stratified by Robson category, (2) assess the extent to which caesarean sections were clinically indicated, and (3) identify any variation across different socioeconomic groups.	Brazil	2011-2017	Type of delivery	Robson classification, Human Development Index, maternal age, maternal education, maternal marital status, pregnancy duration, birthweight, type of pregnancy, fetal presentation, onset of labour, previous pregnancies, previous cesarean sections
**Reis et al., 2021**	The objective of the present study is to describe the geographical and temporal distributions of CA prevalences and IM-CA rates in Brazil, from 2012 to 2017.	Brazil	2012-2017	Congenital anomaly	Region, state
**Salim et al., 2021**	This study aimed to evaluate the association between the diagnosis of malformations of the circulatory system (MCSs) at birth and death from MCSs in the first year of life with the Human Development Index (HDI) and the availability of technological human resources for the diagnosis and treatment of MCSs by macroregion in Brazil.	Brazil	2000-2015	Congenital anomaly	Region, Human Development Index, technological and human resources
**Shimamura et al., 2021**	This study aims to evaluate the frequency of late pregnancies in Brazil, the age-specific fertility rate (ASFR) per region, the rates of prematurity and LBW, and their association with advanced maternal age as compared with 20–34-year-old women.	Brazil	1995-2018	Birthweight, maternal age, pregnancy duration	Region, maternal age

**Falcão et al., 2020**	This study aimed to identify the factors associated with TLBW in impoverished Brazilian women.	Brazil	2001-2015	Birthweight, pregnancy duration	Region, maternal marital status, maternal education, number of antenatal care visits, maternal age, sex, birth order, area of residence, household conditions, maternal race/skin color
**Knobel et al., 2020**	The present study aims to address CS rates according to the Robson classification in the five geographic regions of Brazil, to provide evidence to better understand and outline strategies to help reduce the high CS rate in the country.	Brazil	2014-2016	Type of delivery	Region, Robson classification
**Marteleto et al., 2020**	The first is to investigate whether recent trends in live births, fertility rates, and fertility age patterns have changed amid the ZIKV epidemic in Brazil, the country most affected by this public health shock. The second goal is to examine whether changes in live births and fertility were conditioned by educational level and geographic location (state).	Brazil	2014-2016	Maternal age	State, maternal education
**Rudey et al., 2020**	The main objective of this study was to report and analyze CS rates in Brazil from 2014 to 2017 using the Robson classification system and suggest possible measures to address the issue of high CS rates. The secondary objective was to compare CS rates between regions with a high and low human development index (HDI) according to the Robson classification system.	Brazil	2014-2017	Type of delivery	State and municipalities with high and low Human Development Index, Robson classification
**Bessa and Bonatto, 2019**	The purpose of this study is to promote informed choice for women and provide information on safety of place of birth (hospital births compared with home births) in Brazil based on 1 and 5-minute Apgar score distribution.	Brazil	2011-2015	Apgar	Place of birth
**Buratto et al., 2019**	The objective of this study was to analyze the temporal trend of adolescent pregnancy and its associated factors in Brasil between 2006 and 2015.	Brazil	2006-2015	Maternal age	Region, state, maternal age, maternal race/skin color, maternal education, maternal marital status
**Kluthcovsky et al., 2019**	This study aimed to analyze the temporal trend and socioeconomic factors associated with cesarean sections, which occurred in Brazil and its states (UFs) from 2001 to 2015.	Brazil	2001-2015	Type of delivery	Region, state, percentage of illiterate people, average years of schooling, average household income per capita, percentage of population with income less than half a minimum wage per capita, percentage of households with sewage, percentage of households with garbage collection, Human Development Index (HDI)
**Monteiro et al., 2019**	The objective of this paper is to determine the frequency of adolescence pregnancy in both age groups (10-14 and 15-19 years), in	Brazil	2006-2015	Maternal age	Region, Human Development Index

	all five regions of Brasil, and investigate its association with the Human Development Index (HDI) of each region.				
**Paixão et al., 2019**	This study aims to assess the impact of Zika epidemic on the registration of congenital anomalies in infants in Brazil.	Brazil	2010-2017	Congenital anomaly	Region
**Rothstein et al., 2019**	The present study aims to investigate the proportion of cesarean sections, according to skin color (race/ethnicity) and maternal education in different regions of Brazil, from 2007 to 2016.	Brazil	2007-2016	Type of delivery	Region, maternal education, maternal race/skin color
**Shibukawa et al., 2019**	We aimed to analyze the trend and the factors associated with the presence of cleft lip and/or cleft palate in Brazilian neonates.	Brazil	2005-2016	Congenital anomaly	Region, maternal age, maternal education, maternal marital status, pregnancy duration, number of antenatal care visits, type of delivery, sex, Apgar at 1 and 5 minutes, newborn's race/skin color, birthweight
**Cardoso-Dos-Santos et al., 2018**	We carried out a population-based study of live births in spatial and temporal dimensions.	Brazil	2001-2014	Type of pregnancy	Region, state, maternal education, maternal marital status, maternal age, Human Development Index
**Mallmann et al., 2018**	The objective of this study was to analyze evolution as to conducting seven or more prenatal consultations in Brazil in the period 2000-2015, according to maternal education level and race/skin color of the newborn baby.	Brazil	2000-2015	Antenatal care visits	Maternal education, newborn's race/skin color
**Pereira et al., 2018**	To describe the temporal tendency of the congenital malformations of the nervous system in the period from 2010 to 2014 in Brazil and verify if there are differences in the number of cases accorging to region, maternal age, gestational age, type of delivery, sex, and birthweight.	Brazil	2010-2014	Congenital anomaly	Region, sex, type of delivery, maternal age, pregnancy duration, birthweight
**Santana et al., 2018**	The objective of this study was to assess the prevalence of twin pregnancies in Brazil and their maternal and perinatal characteristics using data from the national birth e- Registry.	Brazil	2011-2014	Apgar, birthweight, congenital anomaly, fetal presentation, onset of labor, pregnancy duration, type of delivery, type of pregnancy	Region, state, maternal education, maternal age, maternal marital status, maternal race/skin color, previous pregnancies, previous cesarean sections, number of antenatal care visits, place of birth, type of pregnancy, birth order, fetal presentation, type of delivery, pregnancy duration, Human Development Index
**Nascimento et al., 2017**	This study aimed to describe trends in the prevalence of live newborns weighing 4,000 g according to gestational age strata, in Brazil and in its five regions, in the periods of 2001–2010 and 2012–2014.	Brazil	2001-2014	Birthweight	Region, pregnancy duration
**Sousa and Roncalli, 2017**	This study aimed at investigating the prevalence of live births with orofacial clefts in Brazil from 2009 to 2013, taking into account their	Brazil	2009-2013	Congenital anomaly	Region, state, number of Authorization Forms for Hospital Admittance (AIHs)

	distribution by geographic region and federative units (Brazilian states).				
**Abreu et al., 2016**	We estimated the yearly prevalence and temporal trend in prevalence of CL/P during the 14-year period from 2000 to 2013. Second, we examined time trends in the birth prevalence (CL/P) in the different Brazilian regions.	Brazil	2000-2013	Congenital anomaly	Region
**Anjos and Boing, 2016**	The present study aimed at investigating the factors associated with conducting prenatal visits in Brazil in 2013.	Brazil	2013	Antenatal care visits	Region, municipality, maternal age, maternal education, maternal marital status, newborn's race/skin color, type of pregnancy, pregnancy duration, birthweight
**Marinho et al., 2016**	Aimed to describe the prevalence rates and characterize cases of microcephaly at birth in Brazil, in the period 2000-2015.	Brazil	2000-2015	Congenital anomaly	Region, state, municipal population size, maternal age, maternal education, maternal race/skin color, maternal marital status, number of antenatal care visits, trimester antenatal care started, place of birth, type of delivery, pregnancy duration, type of pregnancy, month of birth, sex, birthweight, other congenital anomalies, Apgar at 1 and 5 minutes
**Rattner and Moura, 2016**	This study describes the temporal trend of births in Brazil, according to the type of delivery (cesarean or vaginal), between the years 2000 and 2010. At the same time, it analyzes the association of cesarean delivery with variables that demonstrate the inadequacy of obstetric practices, in the year 2011.	Brazil	2000, 2005, 2010, 2011	Type of delivery	Region, day of the week and hour of birth, maternal race/skin color, maternal education, maternal marital status, maternal age
**Vaz et al., 2016**	This study aimed at assessing the frequency of teenage pregnancy in Brazil, in the period from 2000 to 2011, in the five macroregions of the country, focusing on two age groups (10-14 and 15-19 years), correlating the above with the human development index (HDI) of each region.	Brazil	2000-2011	Maternal age	Region, Human Development Index
**Barros et al., 2015**	The objective of this study was to describe time trends and differences in cesarean sections among Brazil's geographic areas using data available from the Department of Informatics of the Unified Health System (DATASUS).	Brazil	2000-2011	Type of delivery	Region, municipality, maternal education, maternal race/skin color, maternal age, previous pregnancies, number of health facilities, illiteracy rate, household income per capita, per capita gross product, adult population with low income
**Guimarães et al., 2014**	The present study sought to identify possible associations between per capita consumption of pesticides (exposure) and pregnancy and birth	Brazil	1997-2001	Birthweight, congenital anomaly, pregnancy	Agricultural pesticide consumption per capita

	events (outcome).			duration	
**Schuler-Faccini et al., 2014**	First assessing the burden of disease caused by neural tube defects (NTDs) in Brazil and the impact of interventions already put in place to address the burden	Brazil	2001-2010	Congenital anomaly	None
**Lima et al., 2013**	This work aims to understand the spatial distribution of low birth weight rates in 2009 and their correlation with social and service indicators in Brazilian states.	Brazil	2009	Birthweight	Maternal age, maternal education, number of antenatal care visits, neonatal ICU beds, pregnancy duration, household income
**Matijasevich et al., 2013**	To estimate the prevalence of preterm births in Brazil, macroregions and federation units (UF) for the period 2000 to 2011, and in more than 5,000 municipalities for the year 2011.	Brazil	2000-2011	Pregnancy duration	Region, state, municipality
**Cremonese et al., 2012**	The aim of the present study was to investigate the association between per capita consumption of pesticides and possible adverse events during pregnancy in the Southern Region of Brazil.	South region	1996-2000	Apgar, birthweight, pregnancy duration	Pesticide exposure
**Hofelmann, 2012**	The objective of this study was to describe the temporal trend of cesarean delivery in Brazil and its distribution by region, from 1994 to 2009, based on the observation of data from Sinasc.	Brazil	1994-2009	Type of delivery	Region, state
**Orioli et al., 2011**	Our aim was to verify changes in the SB birth prevalence using the SINASC: Sistema de Informacões sobre Nascidos Vivos (Live Birth Information System) data available on the Internet after the fortification of wheat and corn flours with folic acid began in Brazil, in June 2004.	Brazil	2004-2006	Congenital anomaly	State, municipality
**Paes and Santos, 2010**	The current study proposes to evaluate the quality of birth records and identify patterns of relationships between variables that reflect maternal and infant factors in the birth certificates and allow summarizing the data on live births in the 188 microregions of the Northeast in 2000, based on factor analysis.	Northeast region	2000	Antenatal care visits, birthweight, maternal age, maternal education, maternal marital status, maternal race/skin color, pregnancy duration, type of delivery	None
**Silva et al., 2010**	The objective of the present study was to examine whether the so-called low birth weight paradox exists in Brazil.	Brazil	1995-2007	Birthweight	Region, infant mortality, type of delivery, maternal education, neonatal ICU beds
**Rodrigues et al., 2009**	Determine the prevalence of orofacial clefts in newborns in Brazil from 1998 to 2002 and assess if the social differences could predispose the Brazilian population to orofacial clefts.	Brazil	1998-2002	Congenital anomaly	Region, sex, Human Development Index, infant mortality, income per capita, Gini index
**Silveira et al., 2009**	This study aims to describe the evolution of prematurity in Brazil, regions and capitals, between 1994 and 2005, according to data from SINASC.	Brazil	1994-2005	Pregnancy duration	Region
**Andrade et al., 2008**	This study analyzes inequalities in the proportion of low birth weight	Brazil	2005	Birthweight	Region, municipal population size,

	infants in Brazil in 2005. Inequalities are examined in light of several factors, such as geographic location, municipal population size, and maternal education. The influence of prenatal care in mitigating inequalities is also examined.				pregnancy duration, maternal education, type of delivery, number of antenatal care visits
**Freitas et al., 2006**	To elaborate a diagnosis of the obstetric care in Brazil according to some selected variables: maternal mortality rate, prenatal care, adolescent pregnancy, types of delivery, availability of obstetric beds and causes of maternal mortality.	Brazil	2003	Antenatal care visits, maternal age, type of delivery	Region

Supplementary Table S3. Detailed description of the publications' results

Author, publication year	Primary analyses	Unity of analysis	Geographical aggregation level	Main results
**Wehrmeister et al., 2025**	Descriptive	Individual	NA	We can observe that at least one ANC visit coverage is 90% or higher for all subgroups in every region. Conversely, the coverage of eight or more ANC visits is much lower, and there is a huge gap between the uneducated and the more educated women, especially in the North and Midwest regions. Furthermore, there are inequalities between regions. The coverage of eight or more ANC visits for women with 8–11 years of formal education in the North region is lower than for women with none or 1–3 years of formal education in the South and Southeast regions.
**Souza et al., 2025**	Trend or change, geospatial, descriptive, multivariable	Ecological	Regional	Areas at higher risk for anomalies were identified in the Northeast, Southeast, and Southern regions. The Northeast region had a higher number of clusters (n=31) and higher prevalence of nervous system anomalies compared to the other regions (9.7/10,000 births). The highest risk of anomalies compared to the other areas was found in the state of Paraíba (RR 2.4; p-value<0.001).
**Cruz et al., 2025**	Trend or change, descriptive	Individual	NA	A total of 10.616.025 live births and 1.310 with congenital ophthalmic malformations were evaluated. The prevalence of congenital eye malformations during the study period was 12 cases per 100,000 live births, with microphthalmia being the main specific anomaly. Cesarean delivery, unsatisfactory Apgar score, weight < 2500g and indigenous ethnicity were some of the associations with the occurrence of congenital ophthalmic anomalies.
**Fernandes et al., 2025**	Multivariable	Individual	NA	Of the 26,107,682 live births included in this study, 144,291 (0.6%) had congenital abnormalities. Black mothers had higher odds of having children with congenital anomalies (OR 1.16; CI 95% 1.14–1.19) than white mothers, and those without prenatal appointments had an increased chance of having children with congenital anomalies (OR 1.47; CI 95% 1.39–1.56) compared to those who started prenatal care in the first months of pregnancy. Maternal age, more than 40 years (OR 2.26; 95% CI 2.20–2.33), and multifetal gestation (OR 1.49; 95% CI 1.45–1.54) were factors associated with a greater chance of live births with congenital anomalies.
**Santos et al., 2025**	Trend or change, geospatial, descriptive, multivariable	Ecological	National, regional	A total of 234 municipalities (4.2%) formed high–high spatial clusters, primarily in the South and Southeast, while 431 municipalities (7.7%) formed low–low clusters, mostly in the Northeast (Moran's I = 0.121, 95% CI: 0.107 to 0.135). High–high clusters had a lower median proportion of adolescent mothers (≤ 19 years: 17.4%) and a higher proportion of mothers aged ≥ 35 years (12.9%) compared to low–low clusters (23.5% and 8.7%, respectively; p< 0.001). High– high clusters also had fewer mothers with less than seven years of education (31.0% vs. 45.9%, p < 0.001) and higher rates of preterm births and low birth weight (p< 0.001). The proportion of White newborns was higher in high–high clusters than in low–low clusters (82.8% vs. 13.6%, p < 0.001)
**Sá da Silva et al., 2025**	Descriptive	Ecological	Municipal	The timely initiation of antenatal care was 70.2% for all adolescents (<20 years), but only 55.6% for those under 13 years old; 14.1% of adolescents started antenatal care at 22 weeks or more of gestation (28.3% among those <13 years old). Timely initiation was

				lower in the North region, among Indigenous girls, and those with low education.
**Melkan et al., 2025**	Trend or change, descriptive	Individual	NA	The prevalence of congenital anomalies was 8.0 per 1,000 live births, with variations across different years and regions within the country. The Southeast region of Brazil, with the highest human development index, displayed the highest prevalence of congenital anomalies. The most frequent congenital anomalies were limb deformities (29.7%), neural tube defects (14.7%), and heart defects (11.6%).
**Gomes et al., 2025**	Trend or change, descriptive	Individual	NA	During this period, 37126352 live births were registered in Sinasc, and 309140 live births were reported with congenital anomalies, accounting for approximately 1% of all births, and representing about 24000 per year (prevalence: 83/10 000 live births). Limb defects were the most prevalent anomaly, occurring in 26/10 000 live births, followed by heart defects (11/10 000) and oral clefts (7/10 000). A higher prevalence of congenital anomalies was found among infants born prematurely (202/10 000), with Apgar scores ≤7 at 1 minute after birth (216/10 000) and at 5 minutes (540/10 000), and among those with low birth weight – that is, < 2 500 g – (250/10 000). A higher prevalence of congenital anomalies was observed among low birth- weight infants born to mothers aged ≥35 years (115/10 000), or with a history of ≥2 miscarriages (109/10 000) or in multiple pregnancies (>120/10 000).
**Vieira et al., 2025**	Trend or change, descriptive	Individual	NA	A total of 34,564,430 live births were recorded in the period. National prevalence of orofacial clefts was 6.73/10,000 live births (95% confidence interval [95%CI 6.64; 6.81])). The Southern region had the highest rate in the period for all types of orofacial clefts. The Northeast region and the states of Alagoas and Piauí showed a rising trend in the period for the three types of orofacial clefts. Other regions showed a stationary trend or increases/ decreases in just one type of cleft. Comparing the pre-pandemic period with the pandemic period, there were no significant changes in the prevalence in the Brazilian regions.
**Rocha et al., 2025**	Multivariable, descriptive	Individual	NA	In the subsequent pregnancy, 11.6% of the neonates were preterm, 5.1% had LBW, and 6.5% had macrosomia. The prevalence of preterm birth, LBW, or macrosomia differed according to the region and area of residence, maternal age, education level, and maternal race/ethnicity (Table 1)
**Cerqueira-Silva et al., 2025**	Multivariable	Individual	NA	5.7% of the births were from women who were internal migrants. The offspring of women who migrated to municipalities with equal/higher HDI (80% of migrations), exhibited a decreased risk of preterm births (RR: 0.94, 95% CI: 0.93–0.95), low birth weight (RR: 0.94, 95% CI: 0.92–0.95) and small for gestational age (RR: 0.92, 95% CI: 0.91–0.93), but higher risk of congenital abnormalities (RR: 1.14, 95% CI: 1.10–1.18). The offspring of women who migrated to municipalities with lower HDI had delayed access to healthcare and worse outcomes except for a lower risk of low birth weight (RR: 0.94, 95% CI: 0.92–0.96).
**Rebouças et al., 2025**	Multivariable	Individual	NA	If all women experienced the same rate as White women, 1.7% of preterm births, 7.2% of low birth weight (LBW), 10.8% of small for gestational age (SGA) and 11.8% of early neonatal deaths would have been prevented. Percentages preventable were higher among Indigenous (22.2% of preterm births, 17.9% of LBW, 20.5% of SGA and 19.6% of early neonatal deaths) and Black women (6% of preterm births, 21.4% of LBW, 22.8% of SGA births and 20.1% of early neonatal deaths).
**Cargnin et al., 2024**	Trend or change, descriptive	Ecological/ individual	Regional, states	A total of 25,385,841 live births were analyzed, among women aged 10 to 35 years or older in Brazil, with the highest occurrence of births in the age group of 20 to 34

				years, corresponding to 928.15 per 1,000 women with a downward trend (p≤0.001). Birth rate's spatial distribution analysis by maternal age strata demonstrated a downward trend in almost all Brazilian states between the ages of 10 and 19 and an increase in the ≥35 age group.
**da Silva et al., 2024**	Multivariable	Individual	NA	A total of 17930817 live births were included in the study. Of these, 155 214(8.7/1000) were exposed to syphilis during pregnancy. Maternal syphilis increased the odds of low birth weight (aOR 1.88, 95% CI: 1.85–1.91), small for gestational age (aOR1.53, 95% CI: 1.51–1.56), and preterm birth (aOR 1.35, 95% CI: 1.33–1.37). Higher odds were observed for pregnant women with VDRL titer ≥64 and untreated maternal syphilis when compared to mothers without syphilis. Analysis stratified by prenatal care showed higher odds for all adverse birth outcomes for mothers attending ≤6prenatal appointments.
**de Macedo filho et al., 2024**	Descriptive	Individual	NA	DATASUS recorded 8493 cases of congenital hydrocephalus in the studied period, with a prevalence of 24.28 per 100,000 newborns, mostly linked to spina bifida. Congenital hydrocephalus caused 60.83 13.98 neonatal deaths per year, with the highest rate among 32e 36 weeks gestational age. Acquired hydrocephalus led to 1063 infant deaths, whereas congenital hydrocephalus resulted in 3122 deaths, with no clear trend by the years. White infants had the highest mortality. A total of 33,184 shunt procedures were performed, with an average cost of $715.37 per pro- cedure. The mortality model showed no significant effects of cost or professionals'salary, but a significant effect of LOS on hospitalization costs was observed
**Moura et al., 2024**	Trend or change, descriptive	Ecological/ individual	National, regions	The most common anomaly in Brazil was supernumerary fingers, classified as ICD-Q69.0, affecting 11,708 children, with a prevalence of 4.02 per 10,000 live births. Mothers aged over 40 years had a 36% higher prevalence of having children with CAUL than mothers under 40 years old (OR = 1.36; 95%CI 1.19-1.56). Newborns weighing ≤ 2,499 g were 2.64 times more likely to have CAUL compared to those weighing ≥ 2,500 g (OR = 2.64; 95% CI 2.55-2.73).
**Ortelan et al., 2024**	Multivariable	Individual	NA	Programme was not associated with all sets of preterm births, moderate-to-late, and severe preterm births, but was associated with a reduction in extreme preterm births (weighted OR: 0.69; 95%CI: 0.63–0.76). This reduction can also be observed among mothers receiving adequate prenatal care (weighted OR: 0.66; 95%CI: 0.59–0.74) and living in better Bolsa Familia management municipalities (weighted OR: 0.56; 95%CI: 0.43–0.74).
**Pinto et al., 2024**	Trend or change, geospatial, descriptive	Ecological/ individual	Regional, states, municipal	A total of 127,022 live births to girls aged 10-14 years were identified during the period, most of whom were Black, 21.1% in common-law or married relationships, with a lower proportion of seven prenatal care appointments and enrollment in the first trimester, a higher proportion of low birth weight and low Apgar score, residing in the North and Northeast. The mean live birth rate for 10-to-14-year-old girls was significantly autocorrelated with space, especially in municipalities of the Midwest and North.
**Polay et al., 2024**	Trend or change, descriptive	Ecological/ individual	National	A total of 47 cases of congenital glaucoma were identified in Brazil during the study period, with the highest incidence between the years of 2018 and 2021. The analysis of the distribution indicated that the states with the highest incidence were São Paulo, followed by Rio Grande do Sul and Pernambuco. Approximately 60% of cases occurred in male individuals, compared with 19 female cases. The ethnic analysis showed the highest incidence among whites and mixed. Regarding the length of pregnancy, statistical differences were

				observed between newborns of different periods of gestation. Infants born from pregnancies lasting between 28 and 31 weeks and 32 and 36 weeks were significant when analyzed with the group between 37 and 41 weeks.
**Silva et al., 2024**	Trend or change, descriptive	Ecological/ individual	National, regional	During the period, 33,699 children were born with orofacial clefts, and 82.1% (27,677) of them were isolated clefts. Regarding these cases, the majority were cleft lip and palate (9,619 or 34.7%), followed by cleft palate (9,442 or 34.1%), and by cleft lip (8,616 or 31.3%).
**Victor et al., 2024**	Trend or change	Ecological	National, regional	A total of 31,887,329 women from all Federative Units of Brazil were included in the study from 2010 to 2020. The Southeast region had the largest proportion of participants, with records from 2015 accounting for 9.5% of the total. Among the women in the study, 49.6% were between the ages of 20 and 29, and the majority (75.5%) had between 8 and 12 years of schooling. The newborns of these women were predominantly male (58.8%) and non-white (59.5%). The study found that there was a trend towards stabilization of increasing proportions of LBW in the North, Northeast, and Centre-West regions between 2010 and 2020. In Brazil and other regions, these tendencies remained stable.
**Zhang et al., 2024**	Trend or change, geospatial, descriptive, multivariable	Ecological/ individual	National, regional	A 10 μg/m3 increment in wildfire-specific PM2.5 was associated with a hazard ratio of 1.047 (95% confidence interval [CI]: 1.032–1.063) for PTB. Stronger associations between wildfire-specific PM2.5 and PTB were observed during earlier pregnancy, among female infants, and pregnant women < 18 years old, in ethnic minorities, with a length of education ≥ 11 years, from low-income or high temperature municipalities, and residing in North/Northeast regions. An estimated 1.47% (95%CI: 1.01%–1.94%) of PTBs were attributable to wildfire-specific PM2.5 in Brazil, increasing from 2010 to 2019. The PTBs attributable to wildfire-specific PM2.5 surpassed those attributed to non-wildfire PM2.5 (0.31%, 95%CI: 0.09%–0.57%).
**Coutinho and Souza, 2024**	Trend or change, descriptive	Ecological	National, regional	Adolescents and young adults, especially those with low education, experienced a marked decrease, while the number of births among older women has been increasing, which is related to changes in age composition and is not fully explained when controlling for trends and seasonality. The effects of the Zika virus and the Covid-19 pandemic were significant, but smaller than speculated, given the pre-existing downward trend.
**Rocha et al., 2024**	Multivariable	Individual	NA	The prevalence of at least one of the vulnerable phenotypes was 16.7% and 0.6% were simultaneously preterm, LBW, and SGA. For those born at term, all phenotypes had a period of growth recovery from 12 mo. For preterm infants, the onset of L/HAZ growth recovery started later at 24 months and the growth trajectories appear to be lower than those born at term, a condition aggravated among children with the 3 phenotypes. Preterm and female infants seem to experience slower growth recovery than those born at term and males.
**Alberton et al., 2023**	Trend or change, descriptive	Ecological/ individual	National, regional	The prevalence of preterm birth in 2011-2021 was 11.1%, stable; the average in the pandemic period 11.3% (95%CI 11.2;11.4%) was similar to that of the base period 11.0% (95%CI 10.6;11.5%); the North region (11.6%) showed the highest proportion between 2011 and 2021; twin pregnancy (56.3%) and pregnant women who had 4-6 prenatal care visits (16.7%) showed an increasing trend (p-value < 0.001); the highest prevalence was observed for extremes of maternal age, pregnant women of Black race/skin color, indigenous women and those with lower level of education.
**Brasil et al., 2023**	Descriptive	Individual	NA	As results, having 7 or more antenatal consultations is a protective factor for alterations in the Apgar score, as well as cesarean delivery and hospital delivery. It was observed that pregnancy care, regarding the studied

				variables, influences the Apgar score.
**Charles et al., 2023**	Trend or change	Individual	NA	During the study period (from 2017 to 2021), about 2.7 million live births were recorded per year, and the missing value for gestational age at delivery was less than 1.5%. The preterm birth prevalence slightly increased during the COVID-19 pandemic compared to the pre-pandemic period (11.32% in 2021 vs 11.09% in 2019, p-value < 0.0001). After adjusting for sociodemographic variables, the OR of preterm births in Brazil has significantly increased, 4% in 2020 (OR: 1.04 [1.03–1.05] 95% CI, p-value < 0.001), and 2% in 2021(OR: 1.02 [1.01–1.03] 95% CI, p-value < 0.001), compared to 2019. At the regional level, the preterm birth pattern in the South, Southeast and Northeast regions show a similar pattern. The highest odds ratio was observed in the South region (2020 vs 2019, OR: 1.07 [1.05–1.10] 95% CI; 2021 vs 2019, OR: 1.03 [1.01–1.06] 95% CI). However, we also observed a significant reduction in the ORs of preterm births in the northern region during the COVID-19 pandemic (2020 vs 2019, OR: 0.96 [0.94–0.98] 95% CI) and (2021 vs 2019, OR: 0.97 [0.95–0.99] 95% CI). Our analysis shows that the pandemic has increased regional variation in the number of preterm births in Brazil in 2020 and 2021 compared to the pre-pandemic years.
**da Silva et al., 2023**	Multivariable	Individual	NA	The frequency of cleft types was statistically significant and different according to geographic origin (inland x coast). For syndromic clefts, the prevalence ratio for cleft lip with/without palate was 3.6 times higher inland (p value= 0.000). The prevalence ratio for cleft lips with/without palate was two times higher inland (p value = 0.000). Logistic regression suggested cleft lip with/without palate was 6.33 more likely to occur in inland regions (p value = 0.000).
**Fernandes et al., 2023**	Trend or change	Ecological	National, regional	The prevalence and infant mortality due to CA has increased in Brazil and in most regions, especially in the Northeast and North. CAs in the musculoskeletal system were the most frequent at birth (29.8/10,000 live births), followed by those in the circulatory system (12.7/10,000 live births), which represented the primary cause of death in this group. The applied linkage technique made it possible to correct the national prevalence of CA by 17.9% during the analyzed period, after retrieving the anomalies reported in SIM, thereby proving to be a good tool to improve the quality of information on anomalies in Brazil.
**Ferreira et al., 2023**	Trend or change, descriptive	Ecological/ individual	National, regional	During the analyzed period, 31,702,562 births were registered in Brazil. Of the valid records, 43.9% were vaginal deliveries and 56.1% were cesarean sections. There was a general increase in cesarean section rates in all Brazilian regions. The North region had the highest percentage of vaginal deliveries (53.5%) and the lowest of cesarean sections (46.5%), while the South, Southeast and Midwest regions had the highest cesarean rates. The study reveals an increase in cesarean rates over the last decade in Brazil, with significant regional differences. The prevalence of cesarean sections in the South, Southeast and Midwest regions raises concerns about the medicalization of childbirth.
**Magalhães et al., 2023**	Trend or change, multivariable	Ecological/ individual	National	We found that 2,1% of newborns had 5th minute Apgar < 7 in 1999 compared with 0,9% in 2018-2019. Multivariate analysis shows that twins and teenage pregnancy are no longer risk factors. Among risk factors, we observed an increase in prematurity, low birth weight and congenital anomalies. An improvement in maternal markers was observed, especially increase in the number of prenatal consultations and schooling.
**Oliveira et al., 2023**	Trend or change, multivariable	Ecological/ individual	National, regional, states	There was an increase in the number of cases of spinal dysraphism in recent years in Brazil with an annual percentage variation of 3.52%. However, the period from

				2005 to 2009 showed a reduction in live births with spinal dysraphism. The regions with the highest incidence were the South and Southeast. The risk increased in mothers born after 1980, older than 30 years and with a high level of education. The risk was increased in live births of whites and blacks, born from double pregnancy and with body weight less than 3,000 g. The absence of prenatal care was associated with a higher incidence.
**Oliveira et al., 2023**	Trend or change, descriptive	Ecological/ individual	Regional	The incidence of CDH is 0.93 cases, while EA is 0.47 and GS is 2.87, all per 10,000 live births. There is an association between all the malformations and premature birth, cesarean delivery, low birth weight, and low Apgar scores. Both EA and GS are associated with maternal age, EA with older, and GS with younger mothers. While EA is associated with multiple pregnancies, GS is associated with fewer years of maternal formal education, single parenting, and a lower number of prenatal consultations. CDH is associated with the male sex and black ethnicity.
**Victor et al., 2023**	Multivariable	Individual	NA	We analyzed 2,632,314 live births in Brazil in 2016, after appropriate adjustments, women living in municipalities with limited availability of fresh foods had a higher chance of having newborns with SGA [OR2nd tertile: 1.06 (1.05–1.07)] and LBW [OR2nd tertile: 1.11 (1.09–1.12)]. Conversely, municipalities with greater availability of ultraprocessed foods had a higher chance of having newborns with SGA [OR3rd tertile: 1.04 (1.02–1.06)] and LBW [OR2nd tertile: 1.13 (1.11–1.16)]. Stratification by race showed that Black and Mixed/Brown women had a higher chance of having newborns with SGA [OR3rd tertile: 1.09 (1.01–1.18)] and [OR3rd tertile: 1.06 (1.04–1.09)], respectively, while Mixed-race women also had a higher chance of having newborns with LBW [OR3rd tertile: 1.17 (1.14–1.20)]. Indigenous women were associated with LGA [OR3rd tertile: 1.20 (1.01–1.45)].
**Zanon et al., 2023**	Trend or change	Ecological	National, regional	The review showed a tendency to decrease the PRR after flour fortification; however, there is no statistical significance between studies. DATASUS data analysis comparing 5 years before and 5 years after mandatory maize and wheat flour fortification demonstrated a rate ratio of 1.05 (95% CI 0.99–1.1; $p = 0.075$). Furthermore, comparing 10 years after additional cassava flour folate fortification, the rate ratio increased to 1.4 (95% CI 1.34–1.45; $p < 0.01$).
**Dallegrave et al., 2022**	Trend or change, descriptive	Ecological/ individual	Regional	Homogeneity was observed regarding the incidence of this malformation in the three states studied - 8.39 cases per 10,000 births, with a slight predominance in Santa Catarina. Concerning the epidemiological profile of the newborns, we observed a 37% higher incidence in males than in females, and the incidence of this malformation occurred more in individuals with low birth weight and premature babies. Regarding the epidemiological profile of mothers of newborns with cleft lip and palate, maternal age showed a bimodal peak, with a 21% higher incidence in women aged under 14 than in those over 35
**Dias et al., 2022**	Descriptive	Individual	NA	Overall and repeated cesarean section rates were 55.1% and 85.3%, respectively. More than 60% of newborns between 37-38 weeks were delivered via cesarean section. Private hospitals in all regions showed the highest cesarean section rates, especially those in the Central-West Region, with more than 80% at all GAs. The overall cesarean section rate was highly correlated with all cesarean section rates of GA subgroups ($r > 0.7$, $p < 0.01$). Regarding repeated cesarean sections, the overall rate was strongly correlated with the rates of 37-38 and 39-41 weeks in public/mixed hospitals, differing from private hospitals, which showed moderate correlations.

**Lucas et al., 2022**	Multivariable	Individual	NA	Results showed that children born in a household where the mother received BF were less likely to have low birth weight (OR 0.93, CI; 0.92-0.94), very low birth weight (0.87, CI; 0.84-0.89), as well as to be born after 37 weeks of gestation (OR 0.98, CI; 0.97-0.99) or 28 weeks of gestation (OR 0.93, CI; 0.88-0.97). There were no significant associations between households where the mother received BF and congenital malformation. On average, the higher the early life exposure to the PBF of the mother, the lower was the prevalence of low birth weight, very low birth weight and congenital malformation of the newborn. No trend was noted for preterm birth.
**Moura et al., 2022**	Multivariable	Ecological	Municipal	The results found suggest that the main causes of LBW for the sample considered refer to the municipality having a lower real GDP per capita, higher Bolsa Família Program coverage, higher proportion of mothers under 19 and over 39 years old and greater proportion of mothers who had up to three prenatal checkups.
**Bessa et al., 2021**	Trend or change, descriptive	Ecological/ individual	National	This research analyzed all live births during the study period, in Brazil. The chance of cryptorchidism in the neonate is more common in women who become pregnant later (≥30 years of age) and with a higher level of education (≥8 years). The risk of cryptorchidism in relation to the federation units is higher in Paraíba, Pernambuco, Sergipe, São Paulo, and Santa Catarina. Regarding the clinical characteristics, the shorter pregnancies, which characterize premature births, are a risk for the appearance of cryptorchidism in the country.
**Bicalho et al., 2021**	Trend or change	Ecological	National, regional	There was a trend of reduction of 3.5% per year in the fertility rate among adolescents ($p<0.05$), in addition to an increasing trend of 6% per year in the proportion of more than six antenatal consultations ($p <0.0001$) and an increasing trend of 6.8% per year in the proportion of caesarean sections ($p<0.0001$).
**Laignier et al., 2021**	Multivariable	Individual	NA	We observed that 157 cases of Down syndrome were reported among 386,571 live births, representing an incidence of 4 in 10,000 live births. Down syndrome was associated with maternal age ≥ 35 years, paternal age ≥ 30 years, the performance of six or more prenatal consultations, prematurity, and low birth weight ($p < 0.05$).
**Martinelli et al., 2021**	Trend or change	Ecological	National, regional	From 2012 to 2019, the proportion of prematurity in Brazil showed a decreasing trend, ranging from 10.87% to 9.95%, with the lowest proportion in 2015, which was 9.77%. Women aged ≥ 45 years and with 4 to 6 prenatal consultations had the highest proportions of prematurity for the period (14.88% to 17.92%) and with an increasing trend. Illiterate and indigenous women, on the other hand, showed a decreasing trend for the period, despite having the highest proportions of prematurity (15.75% to 11.74%).
**Melo, 2021**	Trend or change	Individual	NA	Elective CS before the 39th week rate dropped 2.78 percentage points, which is statistically significant and equivalent to a 24% decrease in this outcome's mean. We also find increases in birth time the percentage of births happening before the 39th week decreased 2.34 percentage points, which is a decrease of 6% in its average.
**Monteiro et al., 2021**	Trend or change	Ecological	National, regional	There was a variation in ASFR in Brazil of 0.78‰ in 1996 to 0.87‰ in 2018 (+11.5%). In the north region, it increased from 1.28‰ to 1.66‰ in 2018. In the northeast region, it increased from 0.72‰ to 1.66‰ (+131%) in 1996–2011,but decreased to 1.31‰ in 2018 (-21% in relation to 2011). When comparing 1996 and 2018, in the southeast region, there was a 22% decrease; in the south region, it was 48.2%; and in the Center-West region, it was 34%; but in the north region, there was a 29.7% increase, and in the northeast region, it was 81.9%. When adding girls who became pregnant aged 13 years and

				gave birth at 14, there was a threefold increase in the rate.
**Paixão et al., 2021**	Trend or change, descriptive	Ecological/ individual	National	The rate of caesarean sections was higher in older and more educated women. Prelabour caesarean sections accounted for more than 54 % of all caesarean deliveries. Women with a previous caesarean section (Group 5) made up the largest group (21.7 %). Groups 6–9, for whom caesarean sections would be indicated in most cases, all had caesarean section rates above 82 %, as did Group 5. The caesarean section rates were higher in municipalities with higher HDI. The general Brazilian caesarean section rate remained stable during the study period.
**Reis et al., 2021**	Trend or change, descriptive	Ecological/ individual	National, regional, states	The prevalence of CA at birth was 81.67/10,000 (95% CI 80.46–82.88), and the IM-CA rate was 27.97/10,000 (95% CI 27.95–28.00) in the studied period. The five CA with the highest rates were polydactyly (9.66/10,000, 95% CI 6.10–9.82), Down syndrome (3.40/10,000, 95% CI 3.41– 5.99), microcephaly (2.92/10,000, 95% CI 2.91–3.12), hydrocephalus (2.72/10,000, 95% CI 2.65–2.90), and spina bifida (2.44/ 10,000, 95% CI 2.43–2.64). São Paulo was the Brazilian state with the highest CA birth rate (119.3/10,000), and Amazonas was the state with the highest IM-CA rate (33.8/10,000).
**Salim et al., 2021**	Trend or change, descriptive	Ecological/ individual	National, regional	The CM rate was 660.8/100 000 LBs, of which 18 444 were due to MCS (diagnosis rate, 38.55/100 000 LBs). Of all Brazilian macroregions, the Southern and Southeastern regions, with the highest HDI values and resources, had the highest MCS diagnosis rates (56.94/100 000 and 62.83/100 000 LBs, respectively). The Northern and Northeastern regions, with the lowest HDI values and resources, had the lowest MCS diagnosis rates (9.77/100 000 and 13.43/100 000 LBs, respectively). The MCS diagnosis rate was 6.4-fold higher in the Southeastern region as compared to the Northern region, but mortality rates were similar in both regions.
**Shimamura et al., 2021**	Trend or change, descriptive	Ecological/ individual	National, regional	The frequency of newborns (NB) of mothers aged ≥35 years increased by 64%. The 35–39-year-old ASFR increased in all regions, except in the northeast. At maternal age ≥35 years, NB increased by 58% between 28 and 36 weeks during the study period. LBW increased between 500 and 1,499 g in 68.7% and between 1,500 and 2499 g in 57% of cases. In 2018, regarding the age range of 20–34 years, the chance of premature delivery was 29% at 35–39 years (OR=1.29), 54% higher at 40–44 years (OR=1.54), and 114% higher at ≥45 years (OR=2.14); while the chance of LBW increased by 28% at 35–39 years (OR=1.28), 56% at 40–44 years (OR=1.56), and 139% at 45 years or older (OR=2.39).
**Falcão et al., 2020**	Multivariable	Individual	NA	Of 8,768,930 term live births analyzed, 3.7% presented TLBW. The highest odds of TLBW were associated with female newborns (OR: 1.49; 95% CI: 1.47–1.50), whose mothers were black (OR: 1.20; 95% CI: 1.18–1.22), had a low educational level (OR: 1.57; 95% CI: 1.53–1.62), were aged ≥35 years (OR: 1.44; 95% CI: 1.43–1.46), had a low number of prenatal care visits (OR: 2.48; 95% CI: 2.42–2.54) and were primiparous (OR: 1.62; 95% CI: 1.60–1.64). Lower odds of TLBW were found among infants whose mothers lived in the North, Northeast and Center-West regions of Brazil compared to those in the South.
**Knobel et al., 2020**	Descriptive	Individual	NA	The overall rate of CS was of 56%. The sample was divided into 11 groups, and vaginal births were more frequent in groups 1 (53.6%), 3 (80.0%) and 4 (55.1%). The highest CS rates were found in groups 5 (85.7%), 6 (89.5%), 7 (85.2%) and 9 (97.0%). The overall CS rate per region varied from 46.2% in the North to 62.1% in the Midwest. Group 5 was the largest obstetric population in the South, Southeast and Midwest, and

				group 3 was the largest in the North and Northeast. Group 5 contributed the most to the overall CS rate, accounting for 30.8% of CSs.
**Marteleto et al., 2020**	Trend or change	Ecological	National, states	Findings suggest a decline in live births that is stratified across educational and geographic lines, beginning approximately nine months after the link between ZIKV and microcephaly was publicly announced. Although declines in total fertility rates were small, fertility trends estimated by age and maternal education suggest important differences in how Zika might have impacted Brazil's fertility structure.
**Rudey et al., 2020**	Trend or change, descriptive	Ecological/ individual	National	A total of 11,774,665 live births were reported in Brazil during 2014 to 2017, most of which were mostly via CS (55.8%). Regions with high human development indexes had significantly higher CS rates than those with low human development indexes. The Robson group (RG) 1 to 4 accounts for 60.2% of live births and 47.1% of all CSs. RG5 was larger than all the other groups and contributed to the highest global rate of CS (31.3%), in addition to being the group who presented the largest growth.
**Bessa and Bonatto, 2019**	Descriptive	Individual	NA	Home birth infants presented significantly higher risk of 0-5 Apgar scores, both in 1 minute (6.4% versus 3%, odds ratio [OR] = 2.2, confidence interval [CI] IC 2–2.4) and in 5minutes(4.8%versus0.4%,OR = 11.5,CI10.5–12.7).Another finding is related to recovery estimates when from an initially bad 1-minute Apgar (< 6) to a subsequently better 5-minute Apgar (> 6). In this scenario, home infants had poorer recovery, Apgar score was persistently < 6 throughout the fifth minute in most cases (71% versus 10.7%, OR 20.4, CI 17–24.6).
**Buratto et al., 2019**	Trend or change	Ecological	National, regional, states	The general proportion of Live Births from adolescent mothers varied from 21.4% in 2006 to 18.1% in 2015. This reduction occurred because of the negative variation observed among mothers aged 15 to 19 years. The indigenous group was the only that did not present a reduction. There was an increase in the proportion of adolescents between four and seven years of formal education and in the proportion of adolescents living with partners. There was a reduction in all Brazilian Regions and in large part of the Federation Units.
**Kluthcovsky et al., 2019**	Trend or change, descriptive	Ecological/ individual	National, regional, states	From the 2010-2012 triennium, all states had caesarean sections greater than 30%. The largest proportions of cesarean sections were observed in the last three years analyzed from 2013 to 2015; the lowest being 34.9% and the highest 66.8%. Between 2007 and 2015 in all states of the Midwest, Southeast and Southern Regions the amount exceeded 50%. All states showed a significant tendency for cesarean sections to increase over time. There was a positive and significant correlation between the number of cesarean sections and per capita income, households with garbage collection and the Human Development Index, and there was a negative correlation for the percentage of the population with incomes below half one minimum wage.
**Monteiro et al., 2019**	Trend or change, descriptive	Ecological/ individual	National, regional	There was a reduction in the percentage of live births (LB) from adolescent mothers (10 to 19 years old) in Brasil by 13.0% over the last ten years. This decline was observed in all Brazilian regions among mothers aged 15 to 19 years. The number of LB increased by 5.0% among mothers aged 10 to 14 years in the North and decreased in the other regions, with higher rates in the South (18.0%). The specific fertility rate for the 15-19-year-old group decreased from 70.9/1,000 to 61.8/1,000 in the period. The proportion of LB is inversely associated with the HDI, except in the Northeast (the lowest HDI in the country), where there was a significant reduction (18.0%) among mothers aged 15-19 and 2% among those aged 10-14 years.
**Paixão et al.,**	Trend or	Ecological	National,	We found that immediately after the interruption point,

**2019**	change		regional	there was a great increase in the notification rate of congenital anomalies of 14.9/10,000 live births in the brain and eye group and of 5.2/10,000 live births in the group not related with brain or eye malformations. This increase in reporting was in all regions of the country (except in the South) and especially in the Northeast. In the period "post-Zika event", unlike the brain and eye group which showed a monthly decrease, the group without brain or eye malformations showed a slow but significant increase (relative to the pre-Zika trend) of 0.2/10,000 live births.
**Rothstein et al., 2019**	Trend or change, descriptive	Ecological/ individual	National, regional	There was an increase in the proportion of cesareans until 2014 (57.07%), with a slight decrease in the years 2015 (55.55%) and 2016 (55.44%). In all regions, the higher the educational level, the greater the proportion of cesarean sections. Regarding skin color, white presented the highest proportion in all regions, especially in the Midwest and South regions. The indigenous population had the lowest proportion of cesareans, despite an increase mainly in the South, pointing to a determination of the region of residence of the mother superior to the other variables, such as race and schooling.
**Shibukawa et al., 2019**	Trend or change, descriptive	Ecological/ individual	National, regional	We analyzed 17,800 live births with presence of cleft lip and/or cleft palate. The Brazilian prevalence rate was 0.51 / 1000 live births, with South and Southeast Regions registering higher rates than the national rate. There was an association with maternal age above 35 years old, with no partner, less than seven prenatal consultations, premature birth and cesarean section. About the factors of the newborn, being male, Apgar less than seven in the 1st and 5th minutes of life, low birth weight and white color were associated.
**Cardoso-Dos-Santos et al., 2018**	Trend or change, geospatial, descriptive	Ecological/ individual	National, regional, states	Twinning rates (TR) averaged 9.41 per 1,000 for the study period and a first-order autoregressive model of time-series analysis revealed a global upward trend over time; however, there were important regional differences. In fact, a Cluster and Outlier Analysis (Anselin Local Moran's I) was performed and identified clusters of high TR in an area stretching from the south of Brazil's Northeast Region to the South Region (Global Moran Index = 0.062, $P < 0.001$). Spearman's correlation coefficient and a Wilcoxon matched pairs test revealed a positive association between Human Development Index (HDI) and TRs in different scenarios, suggesting that the HDI might be an important indicator of childbearing age and assisted reproduction techniques in Brazil. Furthermore, there was a sharp increase of 26.42% in TR in women aged 45 and over during study period.
**Mallmann et al., 2018**	Trend or change	Ecological	National	We analyzed approximately 48 million births; the proportion of seven or more prenatal consultations increased nationally (from 46.0% to 66.9%) in all groups analyzed; the relative difference between the extremes of education level ranged from 3.0 to 2.0, while the absolute difference ranged from 53.1 to 47.7 percentage points; the adjusted ratio between White/Black race/skin color was 1.4 in 2000 and 1.2 in 2015.
**Pereira et al., 2018**	Trend or change, descriptive	Ecological/ individual	National, regional	The highest number of malformations occurred through DATASUS in the period from 2010 to 2014 was in the Southeast region, followed by the Northeast.
**Santana et al., 2018**	Descriptive, multivariable	Ecological/ individual	States	Twin pregnancy occurred in 1.13% in Brazil, with a higher prevalence in regions with a higher HDI. It was associated with a complete higher level of education (22.9% versus 16.3% for singles) and maternal age > 35 years (17.5% versus 11.4% for singles). Preterm birth <32 weeks (prevalence ratio-PR 12.13 [11.93 – 12.33]), low birth weight (PR 17.8 [17.6-18.0] for the first and PR 20.1 [19.8-20.3] for the second twin), and low Apgar score (PR 2.9 [2.8-3.0] for the first and PR 2.7 [2.6-2.8] for the second twin) were the most important perinatal

				outcomes associated with twin pregnancies. A 5-minute Apgar score < 7among twins was associated with inadequate prenatal care, extreme preterm birth, vaginal delivery, intrapartum cesarean, and combined delivery.
**Nascimento et al., 2017**	Trend or change	Ecological	National, regional	In Brazil, the prevalence of macrosomic births was of 5.3% (2001–2010) and 5.1% (2012–2014). The rates were systematically higher in the North and Northeast Regions both in the preterm and in term strata. In the preterm stratum, the North Region presented the highest variation in the prevalence of macrosomia (þ 137.5%) when comparing 2001 (0.8%) to 2010 (1.9%). In the term stratum, downward trends were observed in Brazil as a whole and in every region. The trends for 2012–2014 were more heterogeneous, with the prevalence systematically higher than that observed for 2001–2010. The APC in the preterm stratum (2001–2010) showed a statistically significant trend change in the North (APC: 15.4%; 95%CI: 0.6–32.3) and South (APC: 13.5%; 95%CI: 4.8–22.9) regions. In the term stratum, the change occurred only in the North region(APC:-1.5%;95%CI:-2.5–-0.5).
**Sousa and Roncalli, 2017**	Trend or change, descriptive	Ecological/ individual	National, regional, states	The results showed that the average prevalence of oral clefts in the period was 5.86 per 10,000 live births, with differences observed between the federative units and the regions. The correlation between the prevalence of orofacial clefts and the number of medical procedures associated with this anomaly was statistically significant (r=0.94; p <0.001)
**Abreu et al., 2016**	Trend or change	Ecological	National, regional	The overall reported birth prevalence was 4.85 (95% CI, 4.78–4.91) per 10,000 live births. The reported birth prevalence of CL/P increased over this time period, from 3.94 (95% CI, 3.73–4.17) per 10,000 in 2000 to 5.46 (95% CI, 5.20–5.74) per 10,000 in 2013. The temporal trend differed for different Brazilian geographic regions, being confined primarily to the Northeast (4.7% per year; 95% CI, 4.0%–5.5%), North (3.3% per year; 95% CI, 1.8%–4.7%), and Central (2.9% per year; 95% CI, 0.9%–4.9%) regions.
**Anjos and Boing, 2016**	Descriptive, multivariable	Individual	NA	It was found that 2.7% of pregnant women attended no prenatal visit and 63.1% attended 7 or more. The chance to attend 7 or more prenatal visits was higher among pregnant women aged 40 years or more, with 12 years or more of schooling, living with a roommate, living in the South and Southeast regions, who had a triplet or more pregnancy, with gestational age of 42 weeks or more, and who had children with normal birth weight. Significant regional disparities were identified in the prevalence of women with seven or more prenatal visits.
**Marinho et al., 2016**	Trend or change, descriptive	Ecological/ individual	National, regional	The annual average number of microcephaly cases was 164 for the period 2000-2014, whilst in 2015, 1,608 cases were registered (54.6 cases per 100 thousand LB). Higher coefficients were observed among preterm babies (81.7; 95%CI 72.3;92.2), born from black-skinned (70.9; 95%CI 58.5;85.9) or to brown-skinned (71.5; 95%CI67.4;75.8) women, to women aged ≤19 (70.3; 95%CI 63.5;77.8) or ≥40 (62.1; 95%CI 46.6;82.6), with ≤3 years of study (73.4; 95%CI 58.2;92.4) and residents in the Northeast region (138.7; 95%CI 130.9;147.0).
**Rattner and Moura, 2016**	Trend or change, descriptive, multivariable	Ecological/ individual	National, regional	The proportion of caesarean births in the country increased around 40% from 2000 to 2010. Vaginal births were distributed similarly over the days of the week (around 14% for each day) and according to time of day (around 25%), while caesareans were concentrated on weekdays and during the daytime. The proportion of caesareans was lower in the Northern region (42.8%), among the indigenous population (16.2%), among women with no schooling (25.2%) and among single mothers (42.0%), with a tendency to increase in proportion to age and level of schooling. After adjustment, the Center West region had the highest

				proportion of caesarean births with the same associated variables.
**Vaz et al., 2016**	Trend or change, descriptive	Ecological	National, regional	There was a decrease in the percentage of live births (LB) from teenage mothers (10-19 years) in Brazil (23.5 % in 2000 to 19.2 % in 2011). This reduc- tion was observed in all Brazilian macroregions in the group of mothers aged 15 to 19 years. The number of LB increased by 5.0% among mothers aged 10-14 years (increase in the North and Northeast and decline in the other macroregions). The proportion of LB shows an inversely proportional trend to HDI score, with the Southeast having the highest HDI and the lowest proportion of LB to teenage mothers in the country.
**Barros et al., 2015**	Trend or change, descriptive, multivariable	Ecological/ individual	National, regional, health district	Cesarean rates increased markedly, from 37.9% in 2000 to 53.9% in 2011. Preliminary results from 2012 showed a rate of 55.8%, with the richest geographic areas showing the highest rates. Rates at the municipal level varied from 9%–96%. Cesareans were more common in women with higher education, white skin color, older age, and in primiparas. In the ecological analyses, the number of health facilities per 1 000 population was strongly and positively correlated with cesarean rates, with an increase of 16.1 percentage points (95% Confidence Interval [95%CI] = 4.3–17.8) for each facility. An increase of 1 percentage point in the poverty rate was associated with a decline of 0.5 percentage point in cesarean rates (95%CI = 0.5–0.6).
**Guimarães et al., 2014**	Descriptive	Ecological	States	The results show an association between pesticide consumption and premature births, and pesticide consumption and low birth weight, although the latter could also be a confounding effect.
**Schuler-Faccini et al., 2014**	Trend or change	Ecological	National	Around 20 % of fetal deaths were related to congenital disorders with approximately 5 % of those being NTDs. For infant mortality, congenital disorders were notified in approximately 15 % of cases, with NTDs present in 10 % of the malformed children. Although statistically significant, the prevalence rate ratio (PRR) for spina bifida in live births was only 0.937 (95 % confidence interval (CI) 0.884–0.994), a decrease of 6.3 % when comparing the pre and post-fortification periods. The impact of fortification seemed to be more visible in fetal deaths due to anencephaly (PRR= 0.727, 95 % CI 0.681–0.777) and for spina bifida (PRR=0.700, 95 % CI 0.507–0.967) with associated decreases of 27.3 and 30 %.
**Lima et al., 2013**	Geospatial	Ecological	States	Higher rates of low birth weight are found in the south/southeastern states (Global Moran: 0.267, p = 0.02). Clusters of the high- high type in the Southeast and of the low-low variety in states in the Amazon region are detect- ed. The spatial inequality of low birth weight reflects the socio-economic conditions of the states.
**Matijasevich et al., 2013**	Trend or change, descriptive	Ecological/ individual	National, regional	Official prevalence of preterm births in Brazil was between 6% and 7% for the 2000-2011 period according to the SINASC system, while the corrected estimates showed values between 11% and 12%. In 2011, preterm birth prevalence was only 15% below the estimated.
**Cremonese et al., 2012**	Descriptive	Individual	NA	Premature birth (gestational age < 22 weeks) and low 1 and 5-minute Apgar score (< 8) in both boys and girls showed a significantly higher PR in the upper quartile of pesticide consumption. No significant differences were observed for low birth weight.
**Hofelmann, 2012**	Trend or change	Ecological	National, regional, states	Cesarean section proportion during the analyzed time ranged from 23.3% to 55.7% in Northeast in 1994 and in Southeast in 2008, respectively; in the whole country the proportion ranged from 32.0 to 48.4%; trend increase was observed for all regions except Midwest.
**Orioli et al., 2011**	Trend or change	Ecological	National, states, municipal	The reduction in spina bifida birth prevalence in 2006 was 39% (O/E 5 0.61; 95% confidence interval [CI], 0.55-0.67), and 40% (O/E 5 0.60; 95% CI, 0.53 0.68), after adjusting for state birth number. This reduction was

				significant (p < 0.0001), and heterogeneous among states (X2 5 72.96; p < 0.0001).
**Paes and Santos, 2010**	Multivariable	Ecological	Microregion	In general, regional distribution of birth coverage suggests an increase in the southern States of the Northeast. Quality of completion of variables in the microregions of the Northeast was considered satisfactory. In the factor analysis, data for the variables were reduced to two factors: favorable and unfavorable to delivery.
**Silva et al., 2010**	Trend or change, descriptive	Ecological	National, regional, states	In Brazil, LBW rate trends were non-linear and non-significant: the rate dropped from 7.9% in 1995 to 7.7% in 2000, then increased to 8.2% in 2003 and remained nearly steady thereafter at 8.2% in 2007. However, trends varied among Brazilian regions: there were significant increases in the North from 1999 to 2003 (2.7% per year), and in the South (1.0% per year) and Central-West regions (0.6% per year) from 1995 to 2007. For the entire period studied, higher LBW and lower IMRs were seen in more developed compared to less developed regions. In Brazilian States, in 2005, the higher the IMR rate, the lower the LBW rate (p=0.009); the lower the low schooling rate, the lower the LBW rate (p=0.007); the higher the number of neonatal intensive care beds per 1,000 live births, the higher the LBW rate (p=0.036).
**Rodrigues et al., 2009**	Descriptive	Ecological/ individual	Municipal	The results showed that the mean prevalence of orofacial clefts in Brazil was 0.36 per 1,000 live births. Using Pearson's correlation coefficient, the correlation between cleft rate and social factors was not statistically significant (p > 0.05)
**Silveira et al., 2009**	Trend or change	Ecological	National, regional	At the national level, there was an increase in the preterm birth rate, accompanied by a reduction in the proportion of missing information on gestational age. The Southeast, South, and Central-West regions followed the national trend, while the preterm birth rate fell in the North and Northeast regions. We compared the findings from SINASC with those from population-based studies. The coverage and quality of SINASC has increased over time, but problems with the determination of gestational age still remain, leading to underestimation of preterm birth rates.
**Andrade et al., 2008**	Descriptive	Individual	NA	An analysis of all live births showed a low-birth-weight paradox, namely high percentages in areas with the highest socioeconomic development. The main explanation for these paradoxical findings involves shorter neonatal survival and inadequate recording of premature births in poorer municipalities. Considering at-term live births from non-multiple gestations, inequalities were found in maternal schooling.
**Freitas et al., 2006**	Descriptive	Individual	NA	Maternal mortality rate was 50.83/100000 live births in Brazil. Prenatal care in the Northern and Northeastern regions of the country presented the lowest number of prenatal care appointments (27% of pregnant women with less than 3 appointments). Premature labor was the main diagnosis for hospital admission before delivery. The number of obstetric beds exceeds the population demand throughout the country. The main causes of maternal deaths were direct causes

NA Not applicable

Supplementary Table S4. Detailed description of the metrics of the studies and journals

Author, publication year	Journal (title abbreviation)	Publication type	JIF (2024)	JIF percentile	JIF quartile	JCR category	Citation number	Qualis CAPES classification
**Wehrmeister et al., 2025**	Rev Bras Epidemiol	National	2	48.8	Q3	PUBLIC, ENVIRONMENTAL & OCCUPATIONAL HEALTH	NA	A2
**Souza et al., 2025**	Epidemiol Serv Saude	National	2	48.8	Q3	PUBLIC, ENVIRONMENTAL & OCCUPATIONAL HEALTH	0	A2
**Cruz et al., 2025**	Rev Bras Oftalmol	National	NA	NA	NA	NA	NA	B4
**Fernandes et al., 2025**	BMC Pregnancy Childbirth	International	2.7	78.2	Q1	OBSTETRICS & GYNECOLOGY	0	A1
**Santos et al., 2025**	Int J Environ Res Public Health	International	NA	NA	NA	NA	NA	C
**Sá da Silva et al., 2025**	Cienc Saude Colet	National	1.2	22.3	Q4	PUBLIC, ENVIRONMENTAL & OCCUPATIONAL HEALTH	0	A1
**Melkan et al., 2025**	Plos One	International	2.6	67.8	Q2	MULTIDISCIPLINARY SCIENCES	3	A1
**Gomes et al., 2025**	Rev Panam Salud Publica	International	2.2	56.4	Q2	PUBLIC, ENVIRONMENTAL & OCCUPATIONAL HEALTH	2	A1
**Vieira et al., 2025**	Epidemiol Serv Saude	National	2	48.8	Q3	PUBLIC, ENVIRONMENTAL & OCCUPATIONAL HEALTH	1	A2
**Rocha et al., 2025**	Matern Child Nutr	International	2.6	47.8	Q3	NUTRITION & DIETETICS	0	A1
**Cerqueira-Silva et al., 2025**	Lancet Reg Health Am	International	7.6	95.9	Q1	HEALTH CARE SCIENCES & SERVICES	0	A2
**Rebouças et al., 2025**	Lancet Reg Health Am	International	7.6	95.9	Q1	HEALTH CARE SCIENCES & SERVICES	2	A2
**Cargnin et al., 2024**	Rev Rene	National	0.3	6.0	Q4	NURSING	0	NC
**da Silva et al., 2024**	Int J Gynecol Obstet	International	2.4	70.4	Q2	OBSTETRICS & GYNECOLOGY	3	A2
**de Macedo filho et al., 2024**	World Neurosurg	International	2.1	41.6	Q3	CLINICAL NEUROLOGY	1	A2
**Moura et al., 2024**	Sao Paulo Med J	National	1.6	62.8	Q2	MEDICINE, GENERAL & INTERNAL	1	A2
**Ortelan et al., 2024**	BMC Public Health	International	3.6	82.7	Q1	PUBLIC, ENVIRONMENTAL & OCCUPATIONAL HEALTH	1	A1
**Pinto et al., 2024**	Cienc Saude Colet	National	1.2	22.3	Q4	PUBLIC, ENVIRONMENTAL & OCCUPATIONAL HEALTH	4	A1
**Polay et al., 2024**	Rev Assoc Med Bras	National	1.3	55.0	Q2	MEDICINE, GENERAL & INTERNAL	0	A3
**Silva et al., 2024**	Rev Paul Pediatr	National	2.0	64.1	Q2	PEDIATRICS	4	B1

**Victor et al., 2024**	J Prev	International	1.5	31.6	Q3	PUBLIC, ENVIRONMENTAL & OCCUPATIONAL HEALTH	1	B2
**Zhang et al., 2024**	J Hazard Mater	International	11.3	94.6	Q1	ENGINEERING, ENVIRONMENTAL	2	A1
**Coutinho and Souza, 2024**	Rev Bras Estud Popul	National	NA	NA	NA	NA	1	A1
**Rocha et al., 2024**	Amer J Clin Nutr	International	6.9	91.5	Q1	NUTRITION & DIETETICS	4	A1
**Alberton et al., 2023**	Epidemiol Serv Saude	National	2	48.8	Q3	PUBLIC, ENVIRONMENTAL & OCCUPATIONAL HEALTH	11	A2
**Brasil et al., 2023**	J Health Sci (Londrina)	National	NA	NA	NA	NA	NA	B3
**Charles et al., 2023**	Sci Rep	International	3.9	81.9	Q1	MULTIDISCIPLINARY SCIENCES	7	A1
**da Silva et al., 2023**	Cleft Palate Craniofac J	International	1.9	31.8	Q3	DENTISTRY, ORAL SURGERY & MEDICINE	2	NC
**Fernandes et al., 2023**	Cienc Saude Colet	National	1.2	22.3	Q4	PUBLIC, ENVIRONMENTAL & OCCUPATIONAL HEALTH	10	A1
**Ferreira et al., 2023**	Arq Ciênc Saúde UNIPAR	National	NA	NA	NA	NA	NA	B1
**Magalhães et al., 2023**	Cienc Saude Colet	National	1.2	22.3	Q4	PUBLIC, ENVIRONMENTAL & OCCUPATIONAL HEALTH	0	A1
**Oliveira et al., 2023**	Childs Nerv Syst	International	1.2	18.8	Q4	CLINICAL NEUROLOGY	0	A4
**Oliveira et al., 2023**	J Neonatal Surg	International	NA	NA	NA	NA	2	NC
**Victor et al., 2023**	BMC Pregnancy Childbirth	International	2.7	78.2	Q1	OBSTETRICS & GYNECOLOGY	2	A1
**Zanon et al., 2023**	Childs Nerv Syst	International	1.2	18.8	Q4	CLINICAL NEUROLOGY	1	A4
**Dallegrave et al., 2022**	Rev AMRIGS	National	NA	NA	NA	NA	NA	B4
**Dias et al., 2022**	Cad Saude Publica	National	1.8	41.2	Q3	PUBLIC, ENVIRONMENTAL & OCCUPATIONAL HEALTH	16	A1
**Lucas et al., 2022**	BMC Public Health	International	3.6	82.7	Q1	PUBLIC, ENVIRONMENTAL & OCCUPATIONAL HEALTH	14	A1
**Moura et al., 2022**	Rev Bras Estud Popul	National	NA	NA	NA	NA	1	A1
**Bessa et al., 2021**	J Public Health Res	International	1.8	41.8	Q3	PUBLIC, ENVIRONMENTAL & OCCUPATIONAL HEALTH	3	A4
**Bicalho et al., 2021**	Rev Bras Enferm	National	1.2	23.7	Q4	NURSING	5	A2
**Laignier et al., 2021**	Int J Environ Res Public Health	International	NA	NA	NA	NA	16	C
**Martinelli et al., 2021**	Rev Bras Estud Popul	National	NA	NA	NA	NA	21	A1
**Melo, 2021**	Estud Econ	National	NA	NA	NA	NA	1	A1
**Monteiro et al., 2021**	Rev Assoc Med Bras	National	1.3	55.0	Q2	MEDICINE, GENERAL & INTERNAL	1	A3
**Paixão et al., 2021**	BMC Pregnancy Childbirth	International	2.7	78.2	Q1	OBSTETRICS & GYNECOLOGY	11	A1

**Reis et al., 2021**	J Community Genet	International	1.8	31.7	Q3	GENETICS & HEREDITY	2	A4
**Salim et al., 2021**	Arq Bras Cardiol	National	1.9	44.1	Q3	CARDIAC & CARDIOVASCULA R SYSTEMS	6	A2
**Shimamura et al., 2021**	Rev Assoc Med Bras	National	1.3	55.0	Q2	MEDICINE, GENERAL & INTERNAL	4	A3
**Falcão et al., 2020**	BMC Pregnancy Childbirth	International	2.7	78.2	Q1	OBSTETRICS & GYNECOLOGY	34	A1
**Knobel et al., 2020**	Rev Bras Ginecol Obstet	National	1.4	33.2	Q3	OBSTETRICS & GYNECOLOGY	15	A4
**Marteleto et al., 2020**	Demography	International	3.6	95.0	Q1	DEMOGRAPHY	61	A1
**Rudey et al., 2020**	Medicine (Baltimore)	International	1.4	56.8	Q2	MEDICINE, GENERAL & INTERNAL	59	A3
**Bessa and Bonatto, 2019**	Rev Bras Ginecol Obstet	National	1.4	33.2	Q3	OBSTETRICS & GYNECOLOGY	2	A4
**Buratto et al., 2019**	Rev Assoc Med Bras	National	1.3	55.0	Q2	MEDICINE, GENERAL & INTERNAL	5	A3
**Kluthcovsky et al., 2019**	Mundo Saúde	National	0.0	1.1	Q4	PUBLIC, ENVIRONMENTAL & OCCUPATIONAL HEALTH	5	NC
**Monteiro et al., 2019**	Rev Assoc Med Bras	National	1.3	55.0	Q2	MEDICINE, GENERAL & INTERNAL	18	A3
**Paixão et al., 2019**	Plos Neglect Trop Dis	International	3.4	88.3	Q1	PARASITOLOGY	8	A2
**Rothstein et al., 2019**	Rev Univap	National	0.1	8.5	Q4	MULTIDISCIPLINA RY SCIENCES	NA	B4
**Shibukawa et al., 2019**	Rev Bras Saúde Mater Infant	National	NA	NA	NA	NA	14	A4
**Cardoso-Dos-Santos et al., 2018**	PLoS One	International	2.6	67.8	Q2	MULTIDISCIPLINA RY SCIENCES	7	A1
**Mallmann et al., 2018**	Epidemiol Serv Saude	National	2	48.8	Q3	PUBLIC, ENVIRONMENTAL & OCCUPATIONAL HEALTH	19	A2
**Pereira et al., 2018**	Rev Pesqui Fisioter	National	NA	NA	NA	NA	NA	NC
**Santana et al., 2018**	Biomed Res Int	International	2.6	36.4	Q3	BIOTECHNOLOGY & APPLIED MICROBIOLOGY	10	A2
**Nascimento et al., 2017**	Rev Bras Ginecol Obstet	National	1.4	33.2	Q3	OBSTETRICS & GYNECOLOGY	9	A4
**Sousa and Roncalli, 2017**	Braz Oral Res	National	1.3	31.8	Q3	DENTISTRY, ORAL SURGERY & MEDICINE	34	A2
**Abreu et al., 2016**	Birth Defects Res Part A Clin Mol Teratol	International	NA	NA	NA	NA	24	NC
**Anjos and Boing, 2016**	Rev Bras Epidemiol	National	2	48.8	Q3	PUBLIC, ENVIRONMENTAL & OCCUPATIONAL HEALTH	10	A2
**Marinho et al., 2016**	Epidemiol Serv Saude	National	2	48.8	Q3	PUBLIC, ENVIRONMENTAL & OCCUPATIONAL HEALTH	67	A2
**Rattner and Moura, 2016**	Rev Bras Saúde Mater Infant	National	NA	NA	NA	NA	22	A4
**Vaz et al., 2016**	Rev Assoc Med Bras	National	1.3	55.0	Q2	MEDICINE, GENERAL & INTERNAL	15	A3
**Barros et al.,**	Rev Panam	International	2.2	56.4	Q2	PUBLIC,	29	A1

**2015**	Salud Publica					ENVIRONMENTAL & OCCUPATIONAL HEALTH		
**Guimarães et al., 2014**	Bol Malariol Salud Ambient	International	NA	NA	NA	NA	5	NC
**Schuler-Faccini et al., 2014**	J Community Genet	International	1.8	31.7	Q3	GENETICS & HEREDITY	9	A4
**Lima et al., 2013**	Cienc Saude Colet	National	1.2	22.3	Q4	PUBLIC, ENVIRONMENTAL & OCCUPATIONAL HEALTH	22	A1
**Matijasevich et al., 2013**	Epidemiol Serv Saude	National	2	48.8	Q3	PUBLIC, ENVIRONMENTAL & OCCUPATIONAL HEALTH	20	A2
**Cremonese et al., 2012**	Cad Saude Publica	National	1.8	41.2	Q3	PUBLIC, ENVIRONMENTAL & OCCUPATIONAL HEALTH	19	A1
**Hofelmann, 2012**	Epidemiol Serv Saude	National	2	48.8	Q3	PUBLIC, ENVIRONMENTAL & OCCUPATIONAL HEALTH	NA	A2
**Orioli et al., 2011**	Birth Defects Res Part A Clin Mol Teratol	International	NA	NA	NA	NA	33	NC
**Paes and Santos, 2010**	Cad Saude Publica	National	1.8	41.2	Q3	PUBLIC, ENVIRONMENTAL & OCCUPATIONAL HEALTH	8	A1
**Silva et al., 2010**	Rev Saude Publica	National	2.1	52.4	Q2	PUBLIC, ENVIRONMENTAL & OCCUPATIONAL HEALTH	49	A1
**Rodrigues et al., 2009**	Braz Oral Res	National	1.3	31.8	Q3	DENTISTRY, ORAL SURGERY & MEDICINE	52	A2
**Silveira et al., 2009**	Cad Saude Publica	National	1.8	41.2	Q3	PUBLIC, ENVIRONMENTAL & OCCUPATIONAL HEALTH	19	A1
**Andrade et al., 2008**	Cad Saude Publica	National	1.8	41.2	Q3	PUBLIC, ENVIRONMENTAL & OCCUPATIONAL HEALTH	22	A1
**Freitas et al., 2006**	Einstein	National	0.9	46.2	Q3	MEDICINE, GENERAL & INTERNAL	0	A4

NA Not available **NC** Not classified
